# Multi‐Site Conformational Exchange in the Synthetic Neomycin‐Sensing Riboswitch Studied by ^19^F NMR

**DOI:** 10.1002/anie.202218064

**Published:** 2023-04-28

**Authors:** Jan H. Overbeck, Jennifer Vögele, Felix Nussbaumer, Elke Duchardt‐Ferner, Christoph Kreutz, Jens Wöhnert, Remco Sprangers

**Affiliations:** ^1^ Department of Biophysics I Regensburg Center for Biochemistry University of Regensburg Universitätsstrasse 31 93051 Regensburg Germany; ^2^ Institute for Molecular Biosciences Goethe-University Frankfurt Max-von-Laue-Str. 9 60438 Frankfurt/M. Germany; ^3^ Institute of Organic Chemistry and Center for Molecular Biosciences Innsbruck (CMBI) University of Innsbruck Innsbruck Austria

**Keywords:** Aminoglycosides, ^19^F NMR, NMR Spectroscopy, RNA Dynamics

## Abstract

The synthetic neomycin‐sensing riboswitch interacts with its cognate ligand neomycin as well as with the related antibiotics ribostamycin and paromomycin. Binding of these aminoglycosides induces a very similar ground state structure in the RNA, however, only neomycin can efficiently repress translation initiation. The molecular origin of these differences has been traced back to differences in the dynamics of the ligand:riboswitch complexes. Here, we combine five complementary fluorine based NMR methods to accurately quantify seconds to microseconds dynamics in the three riboswitch complexes. Our data reveal complex exchange processes with up to four structurally different states. We interpret our findings in a model that shows an interplay between different chemical groups in the antibiotics and specific bases in the riboswitch. More generally, our data underscore the potential of ^19^F NMR methods to characterize complex exchange processes with multiple excited states.

## Graphical abstract

Molecular motions are tightly coupled with function. Here we use a unique combination of five different fluorine NMR relaxation methods to reveal the dynamics in three structurally similar riboswitch:ligand complexes. Our data displays unexpectedly complex exchange topologies with up to four structurally different states. Importantly, the associated kinetic and thermodynamics parameters could be accurately determined.

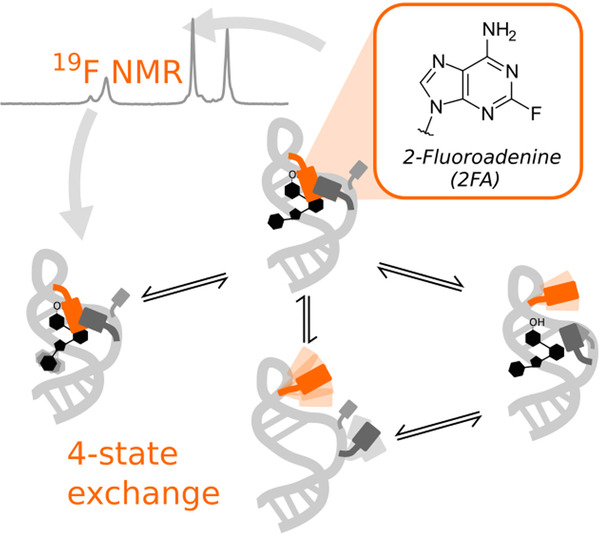

## Introduction

In the last decades, experimental and computational advances have provided ample insights into the structure of a wide range of bio‐molecules. At the same time, our knowledge regarding how these bio‐molecules change shape over time lacks significantly behind,[^1^, ^2^, ^3^] especially when more than two structurally distinct states are simultaneously populated.[^4^, ^5^, ^6^, ^7^, ^8^, ^9^, ^10^, ^11^, ^12^, ^13^] It is clear, however, that these dynamic processes are centrally important for e.g. enzymatic function, molecular recognition, allosteric regulation and bio‐molecular stability. NMR spectroscopy is able to accurately quantify bio‐molecular motions, both in proteins and in RNAs[^14^, ^15^, ^16^, ^17^, ^18^, ^19^] and dedicated experiments that are sensitive to motions at different time‐scales have been introduced.[^2^, ^20^]

**Figure 1:**
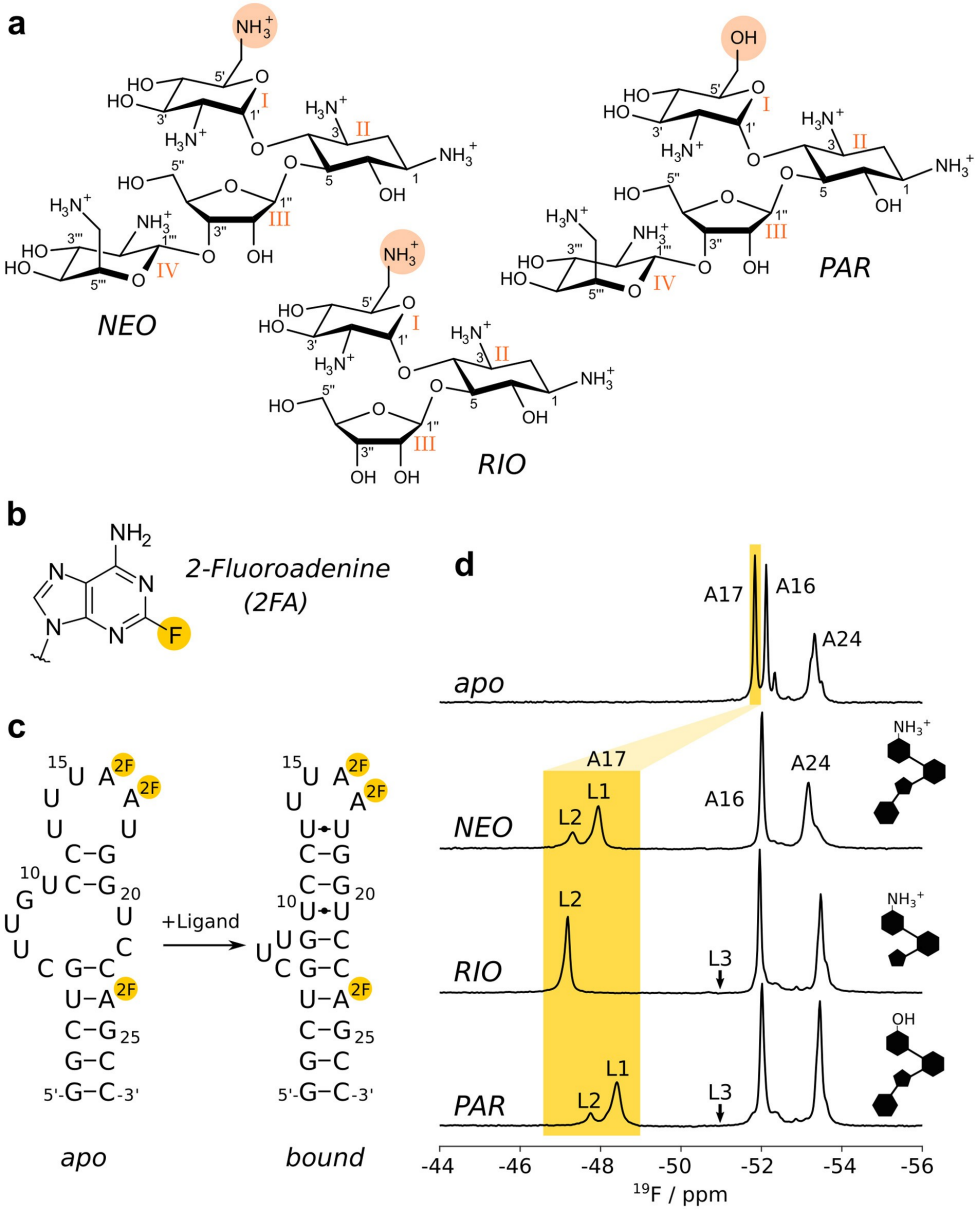
Antibiotic and riboswitch structures. a) Structures of neomycin (NEO), ribostamycin (RIO) and paromomycin (PAR). Ring numbers are indicated with latin numerals (I–IV), the 6′ groups are highlighted in salmon. b) Structure of the 2‐fluoroadenine (2FA) base that is used as an NMR probe in this study. c) Secondary structure of the riboswitch in the apo form (left) and in the presence of a ligand (right), where 2FA‐labeled positions are indicated by spheres. d) ^19^F spectra of the 2FA‐labeled apo riboswitch (top) and of the riboswitch in the presence of NEO, RIO or PAR. The sketches on the right summarize important chemical features of the three ligands: ring I contains an NH_3_^+^ group (NEO and RIO) or an OH group (PAR) and ring IV is either present (NEO and PAR) or absent (RIO). L1, L2 and L3 indicate the chemical shifts of different ligand bound states (see below).

Here, we study the dynamics of a synthetic RNA riboswitch that significantly changes its structure upon interacting with neomycin (NEO) or the closely related ligands ribostamycin (RIO) and paromomycin (PAR) (Figure 1a).^[21]^ The structures of these riboswitch ligand complexes are highly similar.[^22^, ^23^] This is in striking contrast to the difference in their regulatory effectiveness to repress translation initiation, which is highest for NEO, weakened for RIO and completely absent for PAR,^[21]^ despite NEO and PAR differing only in the identity of a single functional group. Interestingly, enhanced dynamics in the ligand‐RNA complexes has been linked with reduced in vivo efficiency.^[23]^

## Results and Discussion

To observe the dynamics of the riboswitch we labeled the RNA simultaneously at positions 16, 17 and 24 with 2‐fluoroadenine by in vitro transcription (Figure 1b, c).[^24^, ^25^] In the apo‐state the ^19^F NMR spectrum displays, as expected, three strong resonances that we assigned through mutagenesis (Figure 1d, Figure S1). In presence of NEO, RIO or PAR, the A17 ^19^F resonance is shifted significantly downfield (Figure 1d). This large chemical shift perturbation is in agreement with the known structures where the base of A17 stacks directly on top of the ligand and acts as a lid of the ligand‐binding pocket.[^22^, ^23^] Interestingly, in presence of saturating (as judged by 1D NMR spectra and CEST experiments) amounts of the NEO and PAR ligands two resonances for A17 are observed. Both resonances are distinct from the resonance in the apo state and thus correspond to two ligand bound states (referred to as ligand states L1 and L2; Figure 1d). This finding reveals the presence of at least two structurally different conformations in the NEO and PAR bound riboswitches that are in slow exchange on the NMR timescale, i.e. the exchange rate *k*_ex,L1‐L2_=*k*_L1→L2_+*k*_L2→L1_ is much smaller than the chemical shift difference Δ*ν*_L1L2_=|*ν*_L1_–*ν*_L2_|=Δ*ω*_L1L2_/2π. As NEO and PAR both contain an aminoglycoside unit (ring IV) that is absent in RIO, we suggest that this unit can adopt two conformations with respect to A17, one where it is in proximity to A17 (that we term state L1), and one where it is remote (NEO or PAR) from A17 or absent (RIO) from the ligand (that we term state L2) (Figure 1d).

**Figure 2:**
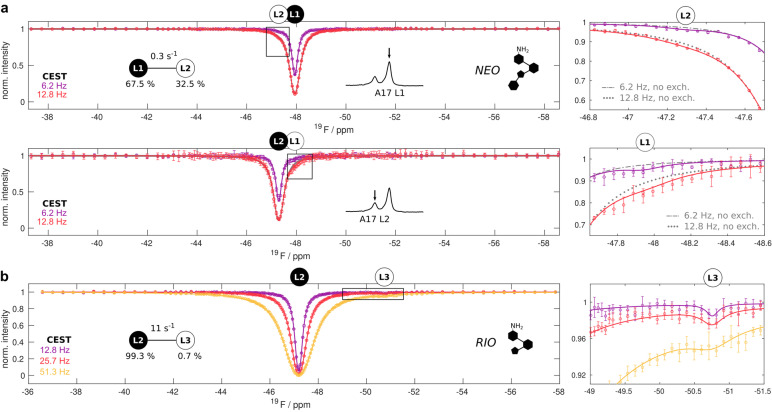
Slow two‐site exchange in the NEO‐ and RIO‐bound riboswitches. a) CEST profiles of the L1 and L2 states of the NEO‐bound riboswitch at 303 K and at B1 fields of 6.2 Hz and 12.8 Hz. The best fit to the data is shown as a solid line for a two‐site exchange process between L1 and L2 (Table S2). The analyzed resonances (top: L1, bottom: L2) are indicated above the CEST profiles and with an arrow in the regions of the indicated 1D spectra (see also Figure 1d). The right panels show zooms of the CEST profiles that are indicated with boxes in the left panel and where the data deviates from the expected CEST profile in the absence of exchange (no exch., grey). b) CEST profiles of the L2 state of the RIO‐bound riboswitch at 303 K and at B_1_ fields of 12.8 Hz, 25.7 Hz and 51.3 Hz. The best fit to the data is shown as a solid line for a two‐site exchange process between L2 and L3 (Table S3). The resonance positions of L2 and L3 are indicated above the CEST profiles. The right panel shows an enlargement of the CEST profile that is indicated with a box in the left panel. In all panels the experimental datapoints are shown as circles with error bars that correspond to 1 standard deviation.

To assess the motions in the NEO saturated riboswitch we employed ^19^F relaxation measurements, that have emerged as a valuable approach to study low‐populated states.[^26^, ^27^, ^28^, ^29^, ^30^] We recorded chemical exchange saturation transfer (CEST) experiments, where magnetization is selectively saturated at variable offsets after which the remaining magnetization is detected.[^31^, ^32^] If a nucleus dynamically samples two conformations the selective perturbation of one state (e.g. L1) will influence the magnetization at the other state (e.g. L2). A careful analysis of these data for the NEO bound riboswitch showed slightly decreased intensity levels of the L1 resonance upon saturation of the L2 resonance and vice versa (Figure 2a, right panels). A global fit, based on the CEST profiles for L1 and L2, with a 2‐state exchange model yielded an exchange rate of *k*_ex_=0.29±0.03 s^−1^ and the populations p_L1_=67.5±3.9 % and p_L2_=32.5±3.9 % at 303 K (Figure 2a).

These populations are in excellent agreement with the relative peak intensities of the L1 and L2 states in the 1D ^19^F spectrum (*I*_L1_=68 %, *I*_L2_=32 % for *I*_L1_+*I*_L2_=100 %). It is worth noting that this exchange rate is very slow and as a result, we were not able to detect this process using 2D ^19^F‐^19^F longitudinal exchange spectroscopy (EXSY) experiments.^[33]^ In EXSY experiments cross‐peaks at frequencies (*ω*_L1_, *ω*_L2_) and (*ω*_L2_, *ω*_L1_) build up during a mixing time in which the conformation of the RNA can change. In the case of the NEO saturated riboswitch, the relaxation rates R_1_ of the L1 and L2 resonances (≈2–3 s^−1^) are, however, faster than the exchange rates (0.29 s^−1^), which prevents the buildup of EXSY cross‐peaks to detectable levels. In summary, we conclude that ring IV in NEO exchanges slowly between the L1 state where it directly interacts with A17 and the L2 state where this motif is remote from A17.

We next studied the dynamics in the RIO‐bound riboswitch that lacks a ring IV and thus only displays the L2 resonance (Figure 1d). Interestingly, CEST profiles of this L2 resonance (Figure 2b), reveal an excited state that resonates at −50.8 ppm (that we term state L3) that was not observed in CEST experiments with the NEO riboswitch and that is not directly visible in the 1D NMR spectra. The CEST data can be fit numerically with a 2‐state exchange model with an L3 population of 0.7±1.3 % that exchanges with the 99.3±1.3 % populated L2 state with a rate of *k*_ex_=11±15 s^−1^, where the large uncertainties reflect the small population of L3. To gain additional insights into the L2–L3 exchange process,[^34^, ^35^] we recorded CEST datasets at 293 K, 298 K and 308 K (Figure S2). At 308 K the L2–L3 exchange rate increased to 21±17 s^−1^. At lower temperatures (293 and 298 K), the L3 state was no longer observable. In summary, we conclude that the RIO bound riboswitch samples a low‐populated excited state L3 in which A17 has a chemical shift that is close to the A17 chemical shift in the apo riboswitch. Based on that, we suggest that the lid‐base A17 is partially dissociated from the ligand in the L3 state.

**Figure 3:**
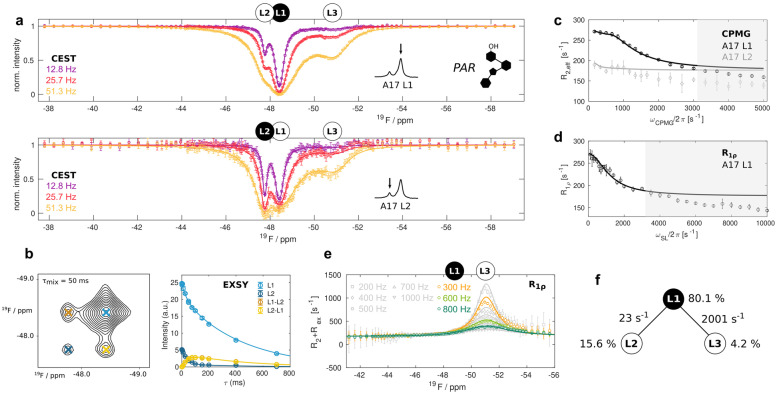
Three‐state exchange in the PAR‐bound riboswitch. a) CEST profiles obtained from analysis of the L1 (top) and the L2 (bottom) resonances. b) Exemplary [^19^F‐^19^F] EXSY spectrum for a mixing time of 50 ms and the dependency of the intensities of the auto (L1, L2) and cross (L1–L2, L2–L1) peaks on the EXSY mixing time. c) CPMG RD profiles of L1 and L2. d) On‐resonance R_1ρ_ RD profile of L1. The grey shaded areas in (c) and (d) were not used in the fitting procedure and indicate additional fast μs timescale motions that are not analyzed here. e) R_2_+R_ex_ contributions derived from off‐resonance R_1ρ_ RD experiments. All data in (a) to (e) were recorded at 303 K and 11.7 T. Data points are shown as open circles with error bars corresponding to 1 standard deviation, the global three‐site exchange fit is shown as solid lines in all panels. f) Fit parameters for the linear L2–L1–L3 model (Table S6).

In the PAR ligand (Figure 1a, d), ring I which directly interacts with the A17 phosphate group contains an −OH group, instead of an −NH_3_^+^ group that is present in the NEO and RIO ligands. The PAR bound riboswitch adopts a structure that is overall identical to the RIO bound aptamer,[^22^, ^23^] but lacks stable A17 : ligand and A17 : C6 stacking interactions^[23]^ and fails to sterically block ribosomal scanning. To illuminate the molecular basis of this functional difference, we collected CEST data of the PAR‐saturated riboswitch at 303 K (Figure 3a). These data clearly reveal that the L1, L2 and L3 states are present at the same time. Based on 2D EXSY experiments (Figure 3b), we conclude that the L1 and L2 states directly exchange with another with a rate on the order of 25 s^−1^, i.e. two orders of magnitude faster than in the L1–L2 exchange in the NEO bound case. Based on initial fits of the CEST data, the exchange rates between states L1/L2 and state L3 must be much faster at 1500–2500 s^−1^. Exchange rates in this range can be accurately quantified using CPMG relaxation dispersion (RD) experiments.[^29^, ^34^] In those experiments the exchange induced line‐broadening in NMR resonances can be suppressed by the application of a variable number of refocussing pulses. Here, we clearly observe a CPMG RD profile for the L1 resonance (Figure 3c). This RD profile cannot directly originate from the exchange process between L1 and L2 as that process is too slow to be observed in the CPMG experiments. We thus conclude that state L1 directly exchanges with state L3.

To obtain additional insights into the L1↔L3 exchange process, we recorded on‐ and off‐resonance R_1ρ_ RD profiles for the L1 state, in which the relaxation rate of a spin‐locked resonance is measured as a function of the spin lock field strength and the spin lock offset, respectively.^[29]^ We clearly observe an exchange to the L3 state (Figure 3d, e).

To extract populations and rates for the three state (L1, L2, L3) exchange process in the PAR saturated riboswitch we used a global fit of the CEST, EXSY, CPMG as well as the on‐ and off‐resonances R_1ρ_ RD data. In the analysis we found that a linear model, in which L2 and L3 do not exchange, was statistically preferred over a full model, where all states exchange directly with each other (see Methods). Notably, the low amplitude CPMG RD dispersion profile observed for L2 (Figure 3c) is caused by the fast exchange between L1 and L3 that is visible in L2 due to the slow exchange between L1 and L2. The linear model fit reveals that the populations of L1, L2 and L3 are 80.1±0.1 %, 15.6±0.1 % and 4.2±0.1 %, respectively, and that the L1↔L2 and L1↔L3 exchange rates are 23±0.4 s^−1^ and 2001±13 s^−1^, respectively at 303 K (Figure 3f, Table S6). The differences in the L1↔L2 exchange rates between the PAR and NEO bound ligands implies that two remote sites in the ligand (the −NH_3_^+^/−OH group in ring I and ring IV) have a direct influence on each other and that antibiotic recognition by the riboswitch is more complex than a simple two‐state binding process.

To obtain information about the thermodynamic parameters of the exchange process that links the three states in the PAR saturated riboswitch we measured ^19^F CEST, CPMG and EXSY experiments at 293 K, 298 K and 308 K (Figure S3). The data from each temperature were globally fit to the linear 3‐state exchange model that we established at 303 K. The retrieved exchange parameters were subsequently fit to Arrhenius and Eyring equations (Figure S4). The L1↔L2 and L1↔L3 exchange processes show an enthalpy‐entropy compensation and are entropically favored (Table S9). This indicates that the L2 and L3 states are less ordered than the L1 state, which is in agreement with a dissociation of ring IV from the RNA (L2) and the opening of the A17 base (L3).

The data above clearly establishes that it is possible to extract accurate exchange parameters from ^19^F experiments for a system that populates three structurally different states. This prompted us to test if other 3‐site exchange processes could also be analyzed. To that end, we extended the 2‐state system of the RIO‐saturated riboswitch (Figure 2b) to a 3‐state system by preparing a sample in which the riboswitch is not fully saturated with RIO. In that system two states correspond to the RIO‐bound riboswitch conformations described above (L2 and L3) and one state corresponds to the lowly populated and thus NMR invisible apo‐riboswitch (A). We recorded ^19^F CEST experiments for the A17 resonance at 293 K, 298 K, 303 K and 308 K (Figure S5). Importantly, the population of the apo riboswitch (A) is markedly visible in the CEST profile of the L2 state. In addition, at 303 K and 308 K the L3 state is clearly visible, in full agreement with the data that was recorded on the riboswitch that was saturated with RIO (Figure 2b, Figure S2). We subsequently fitted the data at 293 K and 298 K using a 2‐state (A↔L2) model and the data at 303 K and 308 K data using a linear 3‐state (A↔L2↔L3) model. In the latter case, it should be noted that fits using a more complex triangular model (where the L3 and A states can also directly exchange) yielded the same χ_ν_ values, reflecting a known difficulty to distinguish kinetic multi‐state schemes in the presence of slow exchange.[^7^, ^36^, ^37^] At 303 K, we obtained populations of 1.6±0.1 % (state A), 98.2±0.1 % (state L2), respectively, 0.3±0.1 % (state L3), as well as an exchange rate of 22±6 s^−1^ for the L2↔L3 exchange (Figure S5).

Importantly, the relative L2 and L3 populations as well as the L2↔L3 exchange rate are in agreement with the data that we obtained on the saturated RIO riboswitch (Figure 2b). For the exchange between L2 and A states we obtained *k*_ex_=[L]*k*_on_+*k*_off_=88±8 s^−1^, from which we extract an off‐rate for the RIO ligand of ≈1.4 s^−1^. Both the population of the apo riboswitch (A), as well as the exchange rate between the A and L2 states increase with temperature (Figure S5), in line with an entropy gain for the transition state of the dissociating ligand.

**Figure 4:**
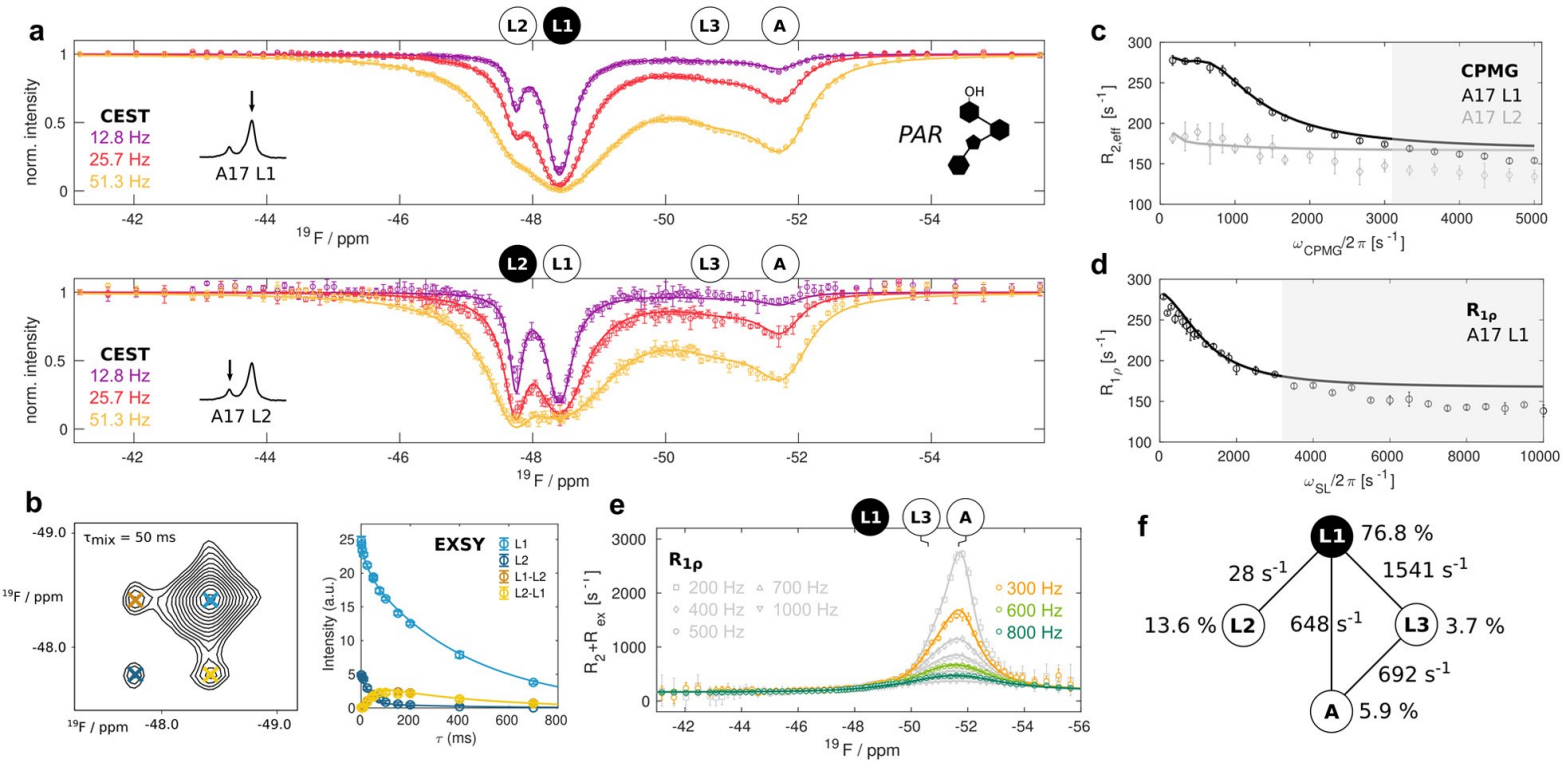
A four‐state exchange in the unsaturated PAR‐bound riboswitch. a) CEST profiles obtained from analysis of the L1 (top) and the L2 (bottom) resonances. b) Exemplary [^19^F–^19^F] EXSY spectrum for a mixing time of 50 ms and the dependency of the intensities of the auto (L1, L2) and cross (L1–L2, L2–L1) peaks on the EXSY mixing time. c) CPMG RD profiles of L1 and L2. c) CPMG RD profile of L1 and L2. d) On‐resonance R_1ρ_ RD profile. The grey shaded areas in (c) and (d) are not used in the fitting procedure and indicates additional fast μs timescale motions that are not analyzed here. e) R_2_+R_ex_ contributions derived from off‐resonance R_1ρ_ RD experiments. All data in (a) to (e) were recorded at 303 K and 11.7 T. Data points are shown as open circles with error bars corresponding to 1 standard deviation, the global four‐site exchange fit is shown as solid lines in all panels. f) Fit parameters for the exchange model (Table S7).

**Figure 5:**
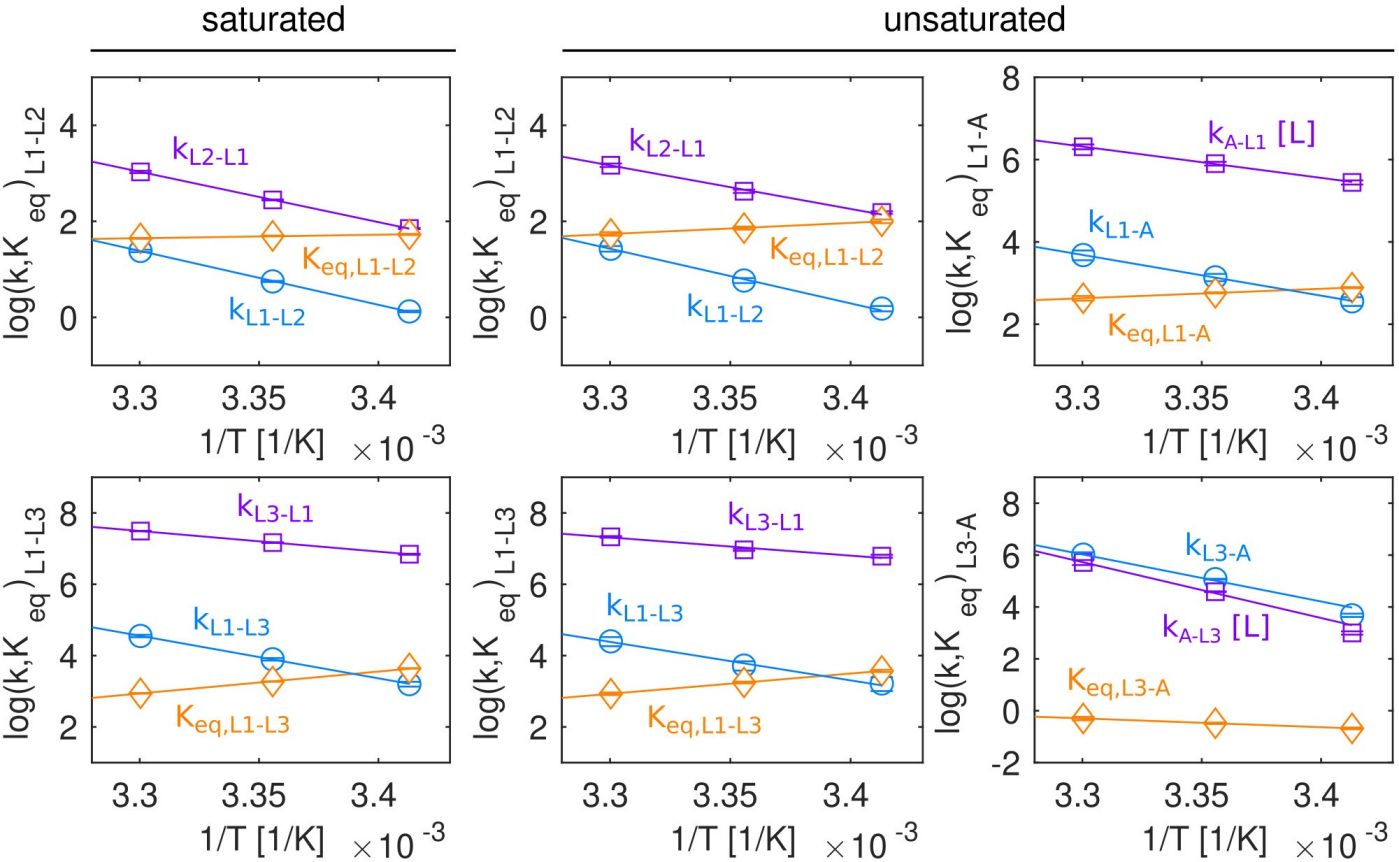
Thermodynamic analysis of the dynamics in the PAR bound riboswitch. Comparison of the temperature dependencies of forward rates, reverse rates and equilibrium constants between different states in the neomycin riboswitch bound to PAR, under saturating (left; Figure 3, Figure S5, Table S9) and non‐saturating (right; Figure 4, Figure S9, Table S9) conditions. Data points at 308 K were omitted in the fit (see supplementary material) and are not shown. Note that the binding of the ligand to the riboswitch is a bimolecular process that depends on the free ligand concentration, where e.g. *k*_ex, L3‐A_=*k*_off_+[L]*k*_on_=*k*_L3‐A_+[L]*k*_A‐L3_.

Finally, we extended our analysis to a system where the riboswitch is only partially saturated with PAR and that thus comprises a total of four structurally different states: the apo riboswitch (A) and the three PAR bound states (L1, L2 and L3). We recorded a full set of ^19^F relaxation experiments (CEST, CPMG, on‐resonance R_1ρ_, off‐resonance R_1ρ_, EXSY) for the A17 resonances at 303 K (Figure 4). In the CEST experiments the resonance of the apo riboswitch is observed upon saturation of the resonances of the states L1 and L2. In line with that, we found a clear increase in the rotating frame relaxation rate R_1ρ_ and its (R_2_+R_ex_) contribution at the offset of the free riboswitch resonance. In contrast, the CPMG and the on‐resonance R_1ρ_ RD experiments are very similar to those recorded on the PAR saturated riboswitch. This indicates that the binding of PAR takes place in an exchange regime that is too slow to be detectable in those RD experiments. In agreement with that, we observe weak EXSY cross peaks between the A and L2 states (Figure S6). We fitted the full set of relaxation data to different 4‐state exchange models and found that the most probable among the 38 possible models is one in which direct exchange between L1 and L2, L1 and L3, L1 and A as well as between L3 and A is considered (4 edges; Figure S10, Table S8, supplementary methods). A global fit of all relaxation data determined that the states A, L1, L2 and L3 are populated to 5.9±0.2, 76.8±0.6, 13.6±0.6 and 3.7±0.2 % respectively (Figure 4f, Table S9). The exchange rates between L1, L2 and L3 (Figure 3) are largely unaffected by the presence of the apo state, as one would expect. We also found that the free riboswitch (A) exchanges with states L1 (648±33 s^−1^) and L3 (691±76 s^−1^) at similar rates (*k*_ex_). The off‐rate of the PAR ligand is thus 10 (from state L1) to 100 (from state L3) times faster than the off‐rate for the RIO ligand (Figure S5) This is in agreement with ITC experiments that reveal that PAR binds weaker to the 2FA‐labeled riboswitch than RIO does (Figure S7). Theoretically, it is possible to deduct dissociation constants (*K*_D_=[free RNA][free ligand]/[RNA : ligand]) from the populations that we derived here. However, even small uncertainties in the absolute RNA and ligand concentrations in the NMR samples result in very high uncertainties in the extracted affinities, preventing a meaningful extraction of *K*_D_ values from the NMR derived populations. To extract thermodynamic information for the four‐site exchange process, we recorded reduced sets of relaxation data (CEST, CPMG, EXSY) at 293 K, 298 K and 308 K (Figure S8) and used the model deduced from the data at 303 K to fit these data. As observed for the RIO ligand, an Arrhenius/Eyring model reveals that PAR binding is associated with a large entropic penalty (Figure 5, Figure S9, Table S9). In addition, we could support our previous finding, that the population of excited ligand‐bound states is entropically driven and that it shows an enthalpy‐entropy compensation.

## Conclusion

**Figure 6:**
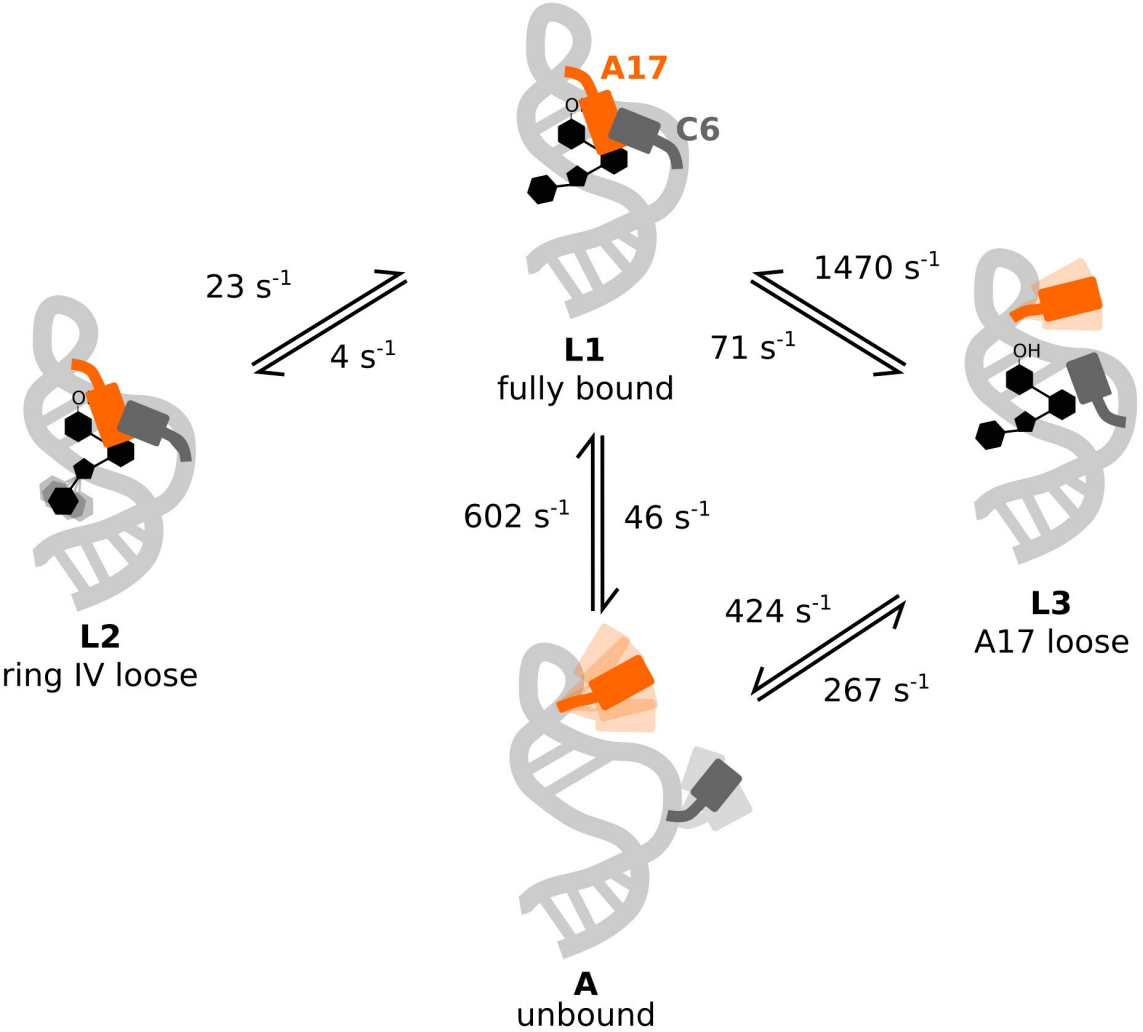
Model of exchange processes in the non‐saturated PAR‐bound neomycin‐sensing riboswitch. In the fully bound state (L1), ring IV of the ligand interacts with U18 and G19, whereas ring I docks onto A17 (orange). This latter interaction is accompanied by the formation of a “paperclip” C6 (dark grey) : A17 stacking interaction.^[38]^ From this state L1, ring IV of the ligand can dissociate such that the “ring IV loose state” (L2) is formed. This state is also present in the RIO‐bound riboswitch (that lacks ring IV), where the stable A17 : C6 stacking has been verified experimentally.^[23]^ Alternatively, A17 can flip out from the L1 state (e.g. in the presence of PAR) such that the “A17 loose state” is formed.

Taken together, we here exploit complementary ^19^F NMR relaxation experiments to obtain insights into the complex dynamic conformational equilibria of the neomycin sensing riboswitch bound to the ligands NEO, RIO and PAR. Our results show a modular influence of the aminoglycoside nature on the conformational energy landscape of the ligand‐bound riboswitches (Figure 6): first, the presence of ring IV (in NEO and PAR) induces a conformational equilibrium between two slowly exchanging states (L1 and L2). Ring IV is unconstrained in the NMR solution structure of the PAR bound riboswitch, but MD simulations indicate, that this ring can contact the phosphate groups of U18 and G19.^[38]^ The L1 and L2 states thus represent sub‐ensembles in which ring IV is either docked to the RNA backbone (L1), or loose (L2). Second, the 6′ group of ring I (−NH_3_^+^ in NEO and RIO, −OH in PAR) determines the relative stability of A17 lid that can either be closed (L1 and L2) or open (L3). In agreement with our NMR data, previous MD simulations report that A17 can flip out when PAR is bound to the riboswitch. In those MD simulations the A17 flipping‐out event was not observed for the RIO‐bound riboswitches,^[39]^ in agreement with the rarity of this event in the presence of RIO (L3 population <1 %). Experimentally, the flipping out of the A17 base is further supported by the chemical shift of the L3 resonance (Figure 1d) that is close to the resonance of the free state (A), and by the large entropic contribution to the L1−L3 exchange process (Figure 5).

Our data reveal that the L1 and L2 conformations are both functional, as the NEO bound riboswitch that contains those two states is able to fully inhibit translation. The reduced efficiency of blocking translation initiation in the RIO bound riboswitch correlates well with the presence of state L3, that is structurally closer to the apo riboswitch than the L1 and L2 states are. In the presence of PAR the state L3 is even more prominent and consequently PAR is not functional in blocking translation. It should be noted, however, that in vivo ribosome blocking is a highly complex process that involves multiple cellular factors. As such, it is not yet possible to correlate the different ligand binding rates or the different structural states unambiguously with function.

In summary, our results shed light on the complex dynamics of ligand‐bound neomycin‐sensing riboswitches and underline the potential of ^19^F NMR to detect and quantitatively study systems with complex exchange topologies. This ^19^F approach complements NMR experiments to study conformational dynamics in nucleic acids that utilise e.g. ^1^H, ^13^C and ^15^N isotopes as reporters.[^40^, ^41^, ^42^, ^43^] The ^19^F methodology is exemplified here for a non‐trivial 4‐state exchange mechanism in a synthetic riboswitch,^[44]^ but is equally applicable to other RNAs, to proteins and to their functional complexes. The sensitivity of the approach is unprecedented as populations as low as 0.3 % and rates as slow as 0.3 s^−1^ could be detected. Based on that we anticipate that future studies on a wide range of bio‐molecules will result in the identification of numerous invisible and lowly populated conformations.

## Conflict of interest

CK is an advisor to and holds an ownership interest in Innotope, a company providing RNA stable isotope labeling products. The remaining authors declare no competing interests.

1

## Supporting information

As a service to our authors and readers, this journal provides supporting information supplied by the authors. Such materials are peer reviewed and may be re‐organized for online delivery, but are not copy‐edited or typeset. Technical support issues arising from supporting information (other than missing files) should be addressed to the authors.

Supporting Information

## Data Availability

The data that support the findings of this study are available from the corresponding author upon reasonable request.
