# Supplementary material for: Multi‐Site Conformational Exchange in the Synthetic Neomycin‐Sensing Riboswitch Studied by 19F NMR
Source: Angew Chem Int Ed Engl. 2023 Apr 28;62(23):e202218064. doi: 10.1002/anie.202218064 (PMC10952710; doi:10.1002/anie.202218064)
Supplement: Supplementary file 1 — Supporting Information [file ANIE-62-0-s001.pdf]

Angewandte Chemie
Eine Zeitschrift der Gesellschaft Deutscher Chemiker

Supporting Information

Multi-Site Conformational Exchange in the Synthetic Neomycin-Sensing Riboswitch Studied by ^{19}F NMR

J. H. Overbeck, J. Vögele, F. Nussbaumer, E. Duchardt-Ferner, C. Kreutz, J. Wöhnert*, R. Sprangers**

Contents

1 Materials and methods **3**

1.1 Synthesis of 2-F-ATP 3
1.2 RNA sample preparation 4
1.3 ITC experiments 4
1.4 NMR spectroscopy 4
1.5 Analysis of NMR data 6
1.5.1 CEST data analysis 6
1.5.2 CPMG data analysis 8
1.5.3 On-resonance and off-resonance $R_{1\rho}$ data analysis 9
1.5.4 EXSY data analysis 10
1.5.5 Estimation of experimental errors 11
1.5.6 Fit optimization procedure 12
1.5.7 Model selection 12
1.5.8 Estimation of exchange parameter uncertainties 13
1.5.9 Thermodynamic analysis 13
1.5.10 Side note on the exchange topologies of n-state systems 14

2 Supplementary Figures **16**

3 Supplementary Tables **27**

4 Supplementary References **31**

1 Materials and methods

1.1 Synthesis of 2-F-ATP

The synthetic access to 2-F-ATP is based on the method published by Santa-Lucia and co-workers [1]. Briefly, 2-F-adenosine (500 mg, 1.87 mmol) was dissolved in anhydrous triethylphosphate (5 mL) at room temperature. After cooling to 0 °C, 2-F-adenosine was transformed into the 5′-monophosphate nucleotide by treatment with $POCl_3$ (2 eq., 349.8 µL, 3,74 mmol) and stirring for 3h at 0 °C. Then, the reaction was quenched at 0 °C by the dropwise addition of sodium hydroxide solution (2M) until pH 4 was reached (controlled by pH paper). The reaction mixture was stirred at 0 °C for one additional hour and then the pH was adjusted to 7 (controlled by pH paper). The reaction mixture was diluted with water and extracted three times with diethyl ether. The monophosphate nucleotide solution was concentrated on a rotary evaporator to a volume of ca. 10 mL and the 2-F-adenosine 5′-monophosphate was then precipitated by the addition of two volumes cold ethanol. After storing the mixture at −20 °C overnight the precipitate was collected by centrifugation at 4 °C, the ethanol/water mixture decanted and the residue dried in high vacuum. The residue was then dissolved in water and further purified by reverse phase chromatography. The final yield was 490 mg (1.41 mmol, 75 %).

The 2-F-ATP was enzymatically prepared by dissolving 490 mg of 2-F-adenosine 5′-monophosphate (1.41 mmol) in 10 mL buffer (50 mM M ATP, 300 mM $MgCl_2$, 300 mM KCl and 150 mM Tris-HCI, pH 7.6) in a 50 mL round bottom flask equipped with a magnetic stirring bar. Then, 824 mg phosphoenolpyruvic acid (monopotassium salt, 2.8 eq., 3.95 mmol) was added and the solution degassed by three Ar-flushing/vacuum cycles. Finally, 10 mg DTT, 1000 U myokinase and 300 U pyruvate kinase were added and the flask was stirred under argon atmosphere at 37 °C. The pH was checked after 1h by using pH paper and adjusted to pH 7.6 with 1M NaOH and/or 1M HCl. This procedure was repeated after an additional hour, then the reaction mixture was allowed to proceed overnight. By using ^{31}P-NMR the reaction progress was monitored and after 16h the reaction was quenched by the addition of 2 volumes of cold ethanol. After storing the mixture at −20 °C overnight the precipitate was collected by centrifugation at 4 °C, the ethanol/water mixture decanted and the residue dried in high vacuum. The residue was then dissolved in water and further purified by reverse phase and weak anion exchange chromatography. The final yield was 350 mg (0.690 mmol, 49 %).

1.2 RNA sample preparation

2F-A modified RNAs were synthesized via *in vitro transcription* using T7 polymerase and synthetic single-stranded DNA templates with a double-stranded promotor region. For transcription 4 mM commercially available cytosine-, guanosine- and uridine-5′-triphosphate (Merck) and 4 mM 2F-adenosine -5′-triphosphate were used. RNA transcripts were purified by preparative urea PAGE according to standard protocols and desalted via PD-10 columns (GE Healthcare). To obtain monomeric hairpin forms, RNAs were folded by heating to 95 °C for 10 minutes followed by injection into 10 equivalents of ice cold water. Concentration and buffer exchange into NMR buffer (25 mM potassium phosphate pH 6.2, 50 mM potassium chloride) was performed using Vivaspin concentrators (MW cutoff 3000 Da, Sartorius).

Sample concentrations varied between 215 and 1200 µM. NMR samples were titrated with 1 to 1.5 equivalents of commercially available neomycin, ribostamycin or paromomycin (Merck) and binding of the ligand by the RNA was followed via the disappearance of fluorine resonances of the free RNA in ^{19}F NMR spectra.

1.3 ITC experiments

Isothermal Titration Calorimetry (ITC) experiments were carried out on a MicroCal iTC200 instrument (Malvern Instruments). Measurements were performed at 37 °C using samples prepared in buffer containing 20 mM Na-cacodylate, 200 mM NaCl, 10 mM $MgCl_2$, 1 mM spermidine, pH 6.8. For measurements with ribostamycin as ligand 10 µM 2F-A modified neomycin riboswitch in the sample cell and 100 µM ribostamycin in the injector syringe were used. Experiments with paromomycin were done using 30 µM RNA and 300 µM ligand solution. After an initial delay of 120 seconds, an initial injection of 0.2 µL was followed by 19 injections of 2 µL at intervals of 180 s and at a stirring speed of 750 rpm. For all measurements, the reference power was set to 11 µcal^{-1}. Thermograms were processed and analyzed using Origin 7.0 software (OriginLab) and thermodynamic parameters were obtained from a curve fit to the data using the one-site binding model. All measurements were repeated three times and the reported K_D values are the average of these experiments.

1.4 NMR spectroscopy

Assignment spectra in figure S1 were recorded on a 600 MHz Bruker NEO NMR spectrometer equipped with a nitrogen cooled ^{1}H/^{19}F [^{13}C,^{15}N] TCI probehead. All ^{19}F EXSY-exchange, CEST, CPMG, on-resonance and off-resonance $R_{1\rho}$ experiments were recorded on a 500 MHz Bruker NEO NMR spectrometer equipped with a triple resonance TCI H&F-C/N-D nitrogen cooled probehead, with the proton coil tuned and matched to the ^{19}F resonance frequency of 470.59 MHz. Data were acquired with a recycle delay of 1.0–1.5 s without ^{1}H

decoupling during the acquisition. The carrier frequency was -51.0 ppm for the 1D spectra and adapted to the resonance of interest for all on-resonance experiments. 90° pulse lengths were calibrated individually for all samples and temperatures and were typically between 12.8 µs and 13.2 µs. Spectra were processed with the NMRpipe software [2] and analyzed with Python and Matlab scripts. Datasets were typically acquired as triplicates to infer experimental errors of all datapoints.

EXSY: ^{19}F- ^{19}F EXSY spectra were acquired as pseudo-3D experiments with an acquisition time of 8.5 ms in the indirect dimension (F1) and mixing times of 1, 2, 5, 10, 25, 50, 75, 100, 150, 200, 400 and 700 ms. Spectra were processed with NMRpipe using exponential window functions with 50 Hz line broadening in both dimensions and converted to plain text using the NMRpipe `pipe2txt.tcl` script. Each spectrum was globally fit with in-house Matlab scripts using four two-dimensional lorentzian functions, in which two parameters for peak positions were used along each dimension and two line widths (Γ_{L1} and Γ_{L2}) were used. Based on these backcalculated spectra the peak volumes were calculated as

$$V = \frac{\pi^2 a \Gamma_x \Gamma_y}{4} \tag{1}$$

where a is the scaling parameter of the lorentzian peak function $\mathcal{L}$, and Γ_x and Γ_y are the full width at half maximum parameters along the direct and indirect dimension:

$$\mathcal{L}(a, x, y, x_0, y_0, \Gamma_x, \Gamma_y) = a \cdot \frac{(\Gamma_x/2)^2}{(x - x_0)^2 + (\Gamma_x/2)^2} \cdot \frac{(\Gamma_y/2)^2}{(y - y_0)^2 + (\Gamma_x/2)^2} \tag{2}$$

CEST: ^{19}F-CEST experiments were acquired as pseudo-2D experiments using 140–156 offset frequencies spaced between 25–200 Hz and centered around the resonance of interest (Figure S11). In brief, a low power pulse is applied at a stepped frequency offset specified by an `fq1list`, followed by an anti-ringing element. 1D spectra were extracted and converted to text format data with the NMRpipe scripts `readROI` and `pipe2txt.tcl` scripts, respectively. Peak intensities were subsequently integrated and analysed with Matlab scripts. CEST fields were calibrated following the determination of B_1 amplitude distributions from Guenneugues [3].

CPMG: ^{19}F-CPMG experiments were acquired with the pulse sequence described previously [4] with a relaxation delay of 6 ms and 20 CPMG frequencies (167, 333, 500, 667, 833, 1000, 1167, 1333, 1500, 1667, 2000, 2333, 2667, 3000, 3333, 3667, 4000, 4333, 4667 and 5000 Hz). Data at frequencies higher than 3 kHz were excluded from the fit, as the observed changes in relaxation rates could be solely attributed to an additional very fast exchange process. To avoid offset effects in the relaxation dispersion, the ^{19}F carrier frequency was set to the maximum of the respective peak in the ^{19}F 1D spectrum.

On-resonance rotating frame relaxation: ^{19}F- on-resonance $R_{1\rho}$ data were collected using the pulse sequence described previously [4] with five different spin lock times T_{SL} (0, 1, 2, 4 and 8 ms) and 31 different spin lock fields (100, 200, 300, 400, 500, 600, 700, 800, 900, 1000, 1200, 1400, 1600, 1800, 2000, 2500, 3000, 3500, 4000, 4500, 5000, 5500, 6000, 6500, 7000, 7500, 8000, 8500, 9000, 9500 and 10000 Hz). As in the CPMG data, data at frequencies higher than 3 kHz were excluded from the fit, as the observed changes in relaxation rates could be solely attributed to an additional very fast exchange process. The ^{19}F carrier frequency was set to the respective peak maximum observed in the one-dimensional ^{19}F spectrum.

Off-resonance rotating frame relaxation: ^{19}F- off-resonance $R_{1\rho}$ experiments were recorded with the pulse sequence described previously (Overbeck et al. 2020) with seven different spin lock times T_{SL} (0, 1, 2, 4, 8, 16 and 32 ms) and 71 different offset frequencies between −3400 Hz and +3400 Hz, spaced between 50–200 Hz and centered around the resonance of interest. Data was acquired for spin lock fields of 50, 100, 150, 200, 300, 400, 600 and 800 Hz.

1.5 Analysis of NMR data

1.5.1 CEST data analysis

In the analysis of CEST data, we extended the procedure introduced by the Kay group [5] from a 2-state exchange to 3-state and 4-state exchange. In this approach, the intensities are numerically calculated using the Bloch-McConnell equations, which are modified by the presence of the CEST field ω_{CEST}. For convenience, we ordered the equations by the components $\{x, y, z\}$ instead of the states $\{a, b, ..., n\}$. The equations can be assembled from the relaxation matrix R, the chemical shift matrix C, the CEST field matrix O and the exchange matrix K. The latter is given by

$$K = \mathbb{1}_3 \otimes \begin{pmatrix} -k_a & k_{ba} & \cdots & k_{na} \\ k_{ab} & -k_b & \cdots & k_{nb} \\ \vdots & \vdots & \ddots & \vdots \\ k_{an} & k_{2n} & \cdots & -k_n \end{pmatrix} \tag{3}$$

where the diagonal elements are given by

$$k_i = \sum_{j=1, j\neq i}^{n} k_{ij} \tag{4}$$

and detailed equilibria are given by

$$k_{ij} = \frac{p_j}{p_i + p_j} \cdot k_{\mathrm{ex},ij} \tag{5}$$

$$k_{ji} = \frac{p_i}{p_i + p_j} \cdot k_{\mathrm{ex},ij} \tag{6}$$

$$k_{\mathrm{ex},ij} = k_{ij} + k_{ji} \tag{7}$$

If all transverse relaxation rates $R_{2,i}$ and longitudinal relaxation rates $R_{1,i}$ are assumed to be identical ($R_{2,j} = R_2$, $R_{1,j} = R_1$), the diagonal relaxation matrix can be written as

$$R = \begin{pmatrix} -R_2 & 0 & 0 \\ 0 & -R_2 & 0 \\ 0 & 0 & -R_1 \end{pmatrix} \otimes \mathbb{1}_n \tag{8}$$

with obvious modifications if any of these rates are treated individually (i.e. if they are fixed or included as separate parameters during minimization), which was not the case in the present work.

The offset δ_i of state i from the low power CEST field ω_{CEST} connects the x and the y components of i as

$$C = \begin{pmatrix} 0 & -1 & 0 \\ 1 & 0 & 0 \\ 0 & 0 & 0 \end{pmatrix} \otimes \begin{pmatrix} \delta_a & 0 & \cdots & 0 \\ 0 & \delta_b & \cdots & 0 \\ \vdots & \vdots & \ddots & \vdots \\ 0 & 0 & \cdots & \delta_n \end{pmatrix} \tag{9}$$

Finally, the CEST field ω_{CEST} enters as

$$O = \begin{pmatrix} 0 & 0 & \omega_{CEST} \\ 0 & 0 & 0 \\ -\omega_{CEST} & 0 & 0 \end{pmatrix} \otimes \mathbb{1}_n \tag{10}$$

For a 2-state exchange $a \rightleftharpoons b$ in the presence of a transverse ω_{CEST} field, the recasted Bloch-McConnell equations can thus be written as a time evolution equation for the vector $\vec{M}' = [M_x^a, M_x^b, M_y^a, M_y^b, M_z^a, M_z^b]^{\mathbf{T}}$ as

$$\frac{\mathrm{d}}{\mathrm{d}t}\vec{M}' = (K + R + C + O) \cdot \vec{M}' = A' \cdot \vec{M}' \tag{11}$$

With explicit consideration of the relaxation back to the equilibrium populations M_{eq}^j, the final equation for the vector $M = [1/2, M_x^a, M_x^b, M_y^a, M_y^b, M_z^a, M_z^b]^{\mathbf{T}}$ is then given by

$$\frac{\mathrm{d}}{\mathrm{d}t}\vec{M} = A \cdot \vec{M} \tag{12}$$

with the evolution matrix A

$$A = \begin{pmatrix} 0 & 0 & 0 & 0 & 0 & 0 & 0 \\ 0 & -R_2 - k_{ab} & k_{ba} & -\delta_a & 0 & 0 & 0 \\ 0 & k_{ab} & -R_2 - k_{ba} & 0 & -\delta_b & 0 & 0 \\ 0 & \delta_a & 0 & -R_2 - k_{ab} & k_{ba} & -\omega_{CEST} & 0 \\ 0 & 0 & \delta_b & k_{ab} & -R_2 - k_{ba} & 0 & -\omega_{CEST} \\ 2R_1 M_{eq}^a & 0 & 0 & \omega_{CEST} & 0 & -R_1 - k_{ab} & k_{ba} \\ 2R_1 M_{eq}^b & 0 & 0 & 0 & \omega_{CEST} & k_{ab} & -R_1 - k_{ba} \end{pmatrix} \quad (13)$$

where the equilibrium magnetizations M_{eq}^i are given by the populations p_i.The intensity of the ground state a after saturating with ω_{CEST} at a given offset δ_{CEST} for a time T_{CEST} can then be directly calculated with a matrix exponential (`expm` in MATLAB) from the starting magnetization $\vec{M}_0 = [E/2, 0, 0, 0, 0, M_z^a, M_z^b]^{\mathbf{T}}$ (where M_z^i are given by p_i) with the projection on the ground state $\vec{M}_{proj} = [0, 0, 0, 0, 0, 1, 0]^{\mathbf{T}}$ as

$$I(\delta_{CEST}) = \vec{M}_{proj} \cdot e^{A \cdot T_{CEST}} \cdot \vec{M}_0 \quad (14)$$

Similar equations with $(n+1) \times (n+1)$ matrices A can be formulated from the above set of equations for $n = 3$ states (yielding a 10×10 matrix) and $n = 4$ states (yielding a 13×13 matrix). Of note, the same equations can be used to calculate the intensity of any other state i by projecting the time-evolved magnetization onto this state. In our case, we used projections on the $L1$ and the $L2$ state for analysis of the NEO- and PAR- bound riboswitches (main text, Figure 3 and Figure 5).

1.5.2 CPMG data analysis

Analysis of CPMG data followed a similar explicit calculation based on the Bloch-McConnell equations. For a 2-state exchange, the full evolution matrices between the π-pulses are given by

$$B = \begin{pmatrix} 0 & 0 & 0 & 0 & 0 & 0 & 0 \\ 0 & -R_2 - k_{ab} & k_{ba} & -\delta_a & 0 & 0 & 0 \\ 0 & k_{ab} & -R_2 - k_{ba} & 0 & -\delta_b & 0 & 0 \\ 0 & \delta_a & 0 & -R_2 - k_{ab} & k_{ba} & 0 & 0 \\ 0 & 0 & \delta_b & k_{ab} & -R_2 - k_{ba} & 0 & 0 \\ 2R_1 M_{eq}^a & 0 & 0 & 0 & 0 & -R_1 - k_{ab} & k_{ba} \\ 2R_1 M_{eq}^b & 0 & 0 & 0 & 0 & k_{ab} & -R_1 - k_{ba} \end{pmatrix} \quad (15)$$

and

$$\widetilde{B} = \begin{pmatrix} 0 & 0 & 0 & 0 & 0 & 0 & 0 \\ 0 & -R_2 - k_{ab} & k_{ba} & \delta_a & 0 & 0 & 0 \\ 0 & k_{ab} & -R_2 - k_{ba} & 0 & \delta_b & 0 & 0 \\ 0 & -\delta_a & 0 & -R_2 - k_{ab} & k_{ba} & 0 & 0 \\ 0 & 0 & -\delta_b & k_{ab} & -R_2 - k_{ba} & 0 & 0 \\ 2R_1 M^a_{eq} & 0 & 0 & 0 & 0 & -R_1 - k_{ab} & k_{ba} \\ 2R_1 M^b_{eq} & 0 & 0 & 0 & 0 & k_{ab} & -R_1 - k_{ba} \end{pmatrix} \tag{16}$$

During the CPMG period T_{CPMG}, magnetization evolves in intervals $\tau_{CPMG} = \frac{T_{CPMG}}{4 \cdot j}$ as

$$\vec{M}(T_{CPMG}) = \left(e^{B \cdot \tau_{CPMG}} e^{\widetilde{B} \cdot \tau_{CPMG}} e^{\widetilde{B} \cdot \tau_{CPMG}} e^{B \cdot \tau_{CPMG}} \right)^j \cdot \vec{M}_0 \tag{17}$$

Effective transverse relaxation rates $R_{2,eff}$ of an observable state i as a function of the CPMG frequency $\omega_{CPMG}/2\pi$ can then be calculated as

$$R_{2,eff} = \frac{1}{T_{CPMG}} \ln \left(\frac{M^i(T_{CPMG})}{p_i} \right) \tag{18}$$

The evolution matrices B and $\widetilde{B}$ show, that transverse and longitudinal magnetization components are not mixed if pulse imperfections and evolution during the pulses are neglected. As a consequence, the matrix equations can be reduced to the evolution of transverse magnetization. For 3-site and 4-site exchange, the matrices can be expanded in a straightforward manner.

1.5.3 On-resonance and off-resonance $R_{1\rho}$ data analysis

For the analysis of rotating frame relaxation data, we initially followed the approach of Trott and Palmer [6]. In brief, for the first-order linear differential equation (11) describing a 2-state exchange (with the CEST field ω_{CEST} replaced by the spin lock field ω_{SL}), the general solution is given by

$$\vec{M}(t) = \sum_{n=1}^{6} e^{\lambda_n t} \vec{l}_n + \vec{S} \tag{19}$$

where $\vec{l}_n$ are proportional to the eigenvectors of the evolution matrix A, and λ_n are the corresponding eigenvalues. The least negative real eigenvalue λ_{lnr} is an estimate for $R_{1\rho}$:

$$R_{1\rho} \approx -\lambda_{lnr} \tag{20}$$

The approach can be extended in a straightforward way to analyze difficult exchange topologies involving more than two states, by computing the eigenvalues of the corresponding evolution matrix. In agreement with the finding, that only two of the six eigenvalues in a 2-site exchange are real [6], we find that three out of nine and four out of twelve eigenvalues

are real for 3-site and 4-site exchange for the exchange regimes encountered in this study. The eigenvalues for an n-state system can be obtained by inverting the corresponding $3n \times 3n$ dimensional matrix. In practice, we used the MATLAB `eig` function to calculate all eigenvalues, and retained the least negative real one. We note that an alternative approach has been formulated, in which the problem is reduced by Schur matrix decomposition to the calculation of characteristic polynomials of 3×3 matrices [7].

In our case, the 3-state and 4-state exchange processes of the PAR-bound riboswitch under saturating and non-saturating conditions include the slow exchange between two substantially populated states, L1 and L2. Under these conditions two eigenvalues become similar in amplitude and the eigenvalue approach breaks down. However, we find that the decay of the spin-locked magnetization for the parameters obtained in our case still hardly deviates from a mono-exponential decay (which is not necessarily the case). We therefore turned to a direct numerical approach [8] and calculated the magnetization for the experimental time points t_i as

$$M(t_i) = \frac{u \cdot e^{At_i} \cdot v}{p_a} \tag{21}$$

where

$$u = \begin{pmatrix} \sin\theta & 0 & \cos\theta \end{pmatrix} \otimes \begin{pmatrix} p_a & p_b & p_c & p_d \end{pmatrix} \tag{22}$$

$$v = \begin{pmatrix} \sin\theta & 0 & \cos\theta \end{pmatrix} \otimes \begin{pmatrix} 1 & 0 & 0 & 0 \end{pmatrix} \tag{23}$$

in which the tip angle is given by the the spin lock frequency ω_{SL} and the offset $\omega_{off} \approx \omega_{\mathrm{L1}}$ as

$$\theta = \arctan \frac{\omega_{SL}}{\omega_{off}} \tag{24}$$

Afterwards, we approximated $R_{1\rho}$ by explicitly fitting the decay to a mono-exponential function $\left(\exp -R_{1\rho}t\right)$. We note that it is also possible to directly compare experimental intensities to numerical back calculations without assuming a mono-exponential decay, and that this approach becomes obligatory in decidedly non-monoexponential decays. In these cases it follows that relaxation of the spin-locked magnetization can not be characterized by a single variable $R_{1\rho}$.

1.5.4 EXSY data analysis

EXSY data were numerically analyzed with a matrix approach. For a 4-site exchange, the autopeak intensities I_{ii} and cross peak intensities $I_{ij,i \neq j}$ after a time T_{EXSY} can be calculated from the starting intensities I_0 with a matrix exponential as

$$\begin{pmatrix} I_{aa} & I_{ba} & I_{ca} & I_{da} \\ I_{ab} & I_{bb} & I_{cb} & I_{db} \\ I_{ac} & I_{bc} & I_{cc} & I_{dc} \\ I_{ad} & I_{bd} & I_{cd} & I_{dd} \end{pmatrix} = e^{E \cdot T_{EXSY}} \cdot \begin{pmatrix} I_0^a & 0 & 0 & 0 \\ 0 & I_0^b & 0 & 0 \\ 0 & 0 & I_0^c & 0 \\ 0 & 0 & 0 & I_0^d \end{pmatrix} \tag{25}$$

where

$$E = \begin{pmatrix} -R_1^a - k_a & k_{ba} & k_{ca} & k_{da} \\ k_{ab} & -R_1^b - k_b & k_{cb} & k_{db} \\ k_{ac} & k_{bc} & -R_1^c - k_c & k_{dc} \\ k_{ad} & k_{bd} & k_{cd} & -R_1^d - k_d \end{pmatrix} \tag{26}$$

In practice, we calculated the full evolution matrix of the 3-site and 4-site exchange, respectively, as exemplified in matrix (15) for a 2-site exchange, and only considered the longitudinal part of the magnetization. Of note, it is not necessary to know the initial intensities of states $k \neq i, j$ not involved in the exchange $i \rightleftharpoons j$ under consideration in order to obtain the autopeak and crosspeak intensities I_{ii}, I_{ij}, I_{ji} and I_{jj}.

1.5.5 Estimation of experimental errors

Experimental errors were determined from replicates. For experimental data points $D_{exp,i}$ of replicate i, the error was calculated as

$$\sigma = \sqrt{\frac{1}{N-1} \sum_{i=1}^{N} \left(D_{exp,i} - \overline{D_{exp}} \right)^2} \tag{27}$$

with the mean experimental value

$$\overline{D_{exp}} = \frac{1}{N} \sum_{i=1}^{N} D_{exp,i} \tag{28}$$

Due to the small replicate size (typically triplicates), errors of individual datapoints can heavily underestimate the true experimental error, which subsequently skews the data fitting. In order to prevent such bias, we introduced minimal errors σ_{min}^{j} for all experiments j, based

on

$$\sigma_{min}^{CEST} = 0.005 \text{ [normalized intensity]} \tag{29}$$

$$\sigma_{min}^{CPMG} = 2\,\mathrm{s}^{-1} \tag{30}$$

$$\sigma_{min}^{EXSY} = 0.01 \times I_{\max} \tag{31}$$

$$\sigma_{min}^{R_{1\rho}^{off}} = 1\,\mathrm{s}^{-1} \tag{32}$$

$$\sigma_{min}^{R_{1\rho}^{on}} = 2\,\mathrm{s}^{-1} \tag{33}$$

$$\tag{34}$$

1.5.6 Fit optimization procedure

Experimental data were numerically fitted with the approaches described above by minimization of a combined χ^2, which sums up the contributions χ_j^2 of the datasets j under consideration

$$\chi^2 = \sum_{j=1} \chi_j^2 \tag{35}$$

with

$$\chi_j^2 = \sum_{i=1}^{m_j} \left(\frac{D_{fit}^{i,j} - D_{exp}^{i,j}}{\sigma_{i,j}} \right)^2 \tag{36}$$

In practice, we used the MATLAB function `fminsearch` to implement the minimization.

1.5.7 Model selection

For exchange scenarios with $n > 2$, the general scheme in which all states directly exchange with each other might not be justified by the experimental data, although it will necessarily yield the same or a lower χ^2, due to the additional parameters k_{ex}^{ij} compared to any reduced model. In order to avoid this caveat of overfitting, we used three criteria (reduced $\chi^2 = \chi_\nu^2$, Akaike information criterion (AIC) and Bayesian information criterion (BIC)) to evaluate, which exchange topology should be chosen to fit the data.

$$\chi_\nu^2 = \frac{\chi^2}{N - K} \tag{37}$$

$$\mathrm{BIC} = N \ln\left(\frac{\chi^2}{N}\right) + K \ln(N) \tag{38}$$

$$\mathrm{AIC} = \begin{cases} N \ln\left(\frac{\chi^2}{N}\right) + 2K & \text{if } N/K \geq 40 \\ N \ln\left(\frac{\chi^2}{N}\right) + 2K + \frac{2K(K+1)}{N-K-1} & \text{if } N/K < 40 \end{cases} \tag{39}$$

where N is the total number of data points and K is the number of fitting parameters in a given model. We then used the differences in AIC and BIC values ($\Delta\mathrm{AIC}_i$, $\Delta\mathrm{BIC}_i$) from different models i to calculate normalized likelihood estimates (weights w_i^{AIC}, w_i^{BIC}) for each model [9, 10], i.e. models that are more likely according to AIC (BIC) have lower AIC (BIC) values and larger AIC (BIC) weights:

$$w_i^{\mathrm{AIC}} = \exp\left(-\frac{1}{2}\Delta\mathrm{AIC}_i\right) \Big/ \sum_{j=1}^{J} \exp\left(-\frac{1}{2}\Delta\mathrm{AIC}_i\right) \tag{40}$$

$$w_i^{\mathrm{BIC}} = \exp\left(-\frac{1}{2}\Delta\mathrm{BIC}_i\right) \Big/ \sum_{j=1}^{J} \exp\left(-\frac{1}{2}\Delta\mathrm{BIC}_i\right) \tag{41}$$

1.5.8 Estimation of exchange parameter uncertainties

To estimate errors in the parameters (populations, exchange rates, etc.) extracted from the fitting of NMR data (CEST, CPMG, etc.) 500 cycles of Monte Carlo simulations were carried out. In each cycle, each data point was randomly varied according to its standard variation, with minimal values given in section 1.5.5. These randomly varied datasets were then numerically fitted, with starting parameters randomly varied around their optimum value. Finally, standard deviations were calculated from the distribution of optimal solutions obtained from all runs.

1.5.9 Thermodynamic analysis

Thermodynamic parameters for the detailed equilibrium between two states i and j were obtained from analysis of the equilibrium constant $K_{\mathrm{eq},ij}$ as well as forward and reverse rates (k_{ij}, k_{ji}) for {CEST, CPMG, EXSY}-datasets between 293 K and 303 K. First, $K_{\mathrm{eq},ij}$ was fit according to

$$K_{\mathrm{eq},ij} = e^{-\Delta G_{ij}/RT} = e^{(T\Delta S_{ij} - \Delta H_{ij})/RT} \tag{42}$$

The values obtained for the change in entropy ΔS_{ij} and in enthalpy ΔH_{ij} were subsequently used as constraints

$$\Delta S_{ij} = \Delta S^{\ddagger i\to j} + \Delta S^{\ddagger j\to i} \tag{43}$$

$$\Delta H_{ij} = \Delta H^{\ddagger i\to j} + \Delta H^{\ddagger j\to i} \tag{44}$$

in the fit equations

$$k_{ij} = \frac{\kappa k_B T}{h} e^{(T\Delta S^{\ddagger i\to j} - \Delta H^{\ddagger i\to j})/RT} \tag{45}$$

$$k_{ji} = \frac{\kappa k_B T}{h} e^{(T\Delta S^{\ddagger j\to i} - \Delta H^{\ddagger j\to i})/RT} \tag{46}$$

where k_B is the Boltzmann constant ($1.38 \times 10^{-23}\,\mathrm{J\,K^{-1}}$) and h is the Planck constant ($6.626 \times 10^{-34}\,\mathrm{J\,s}$), ΔS_{ij} and ΔH_{ij} are the differences in entropy and enthalpy between state i and state j, $\Delta S^{\ddagger i\to j}$ and $\Delta H^{\ddagger i\to j}$ are the entropy and enthalpy differences between state i and the transition state between i and j, $\Delta S^{\ddagger j\to i}$ and $\Delta H^{\ddagger j\to i}$ are the entropy and enthalpy differences between state j and the transition state between i and j, and the transmission coefficient κ was assumed to equal one.

1.5.10 Side note on the exchange topologies of n-state systems

Data analysis of NMR experiments that probe dynamics in the range of microseconds to seconds is based on the assumption, that the exchanging system adopts n distinct states. A state i is characterized by a chemical shift δ_i, a population p_i, other parameters such as relaxation rates R_1^i and R_2^i, and exchange rates $k_{\mathrm{ex},ij} = k_{ij} + k_{ji}$ with any other state j. For $n = 1$, only the ground state (GS) is populated ($p_{GS} = p_a = 1$), for $n = 2$, a single excited state exchanges with the ground state with an exchange rate $k_{\mathrm{ex},ab}$, for $n = 3$ there are two excited states with three exchange rates $k_{\mathrm{ex},ab}$, $k_{\mathrm{ex},ac}$ and $k_{\mathrm{ex},bc}$. In general, for n-site exchange, there will be a total of $n \cdot (n-1)/2$ exchange rates from which it follows directly, that there are $2^{n\cdot(n-1)/2}$ different (not necessarily fully connected) topologies [11]. The number of distinct topologies for distinguishable states (e.g. distinguishable by their chemical shift) without disconnected states can be calculated by graph theory and is equal to the number of labeled, connected graphs. For a graph with n nodes, the number of connected labeled graphs C_n is given by the recurrence relation [12]

$$C_n = \sum_{k=1}^{n-1} \binom{n-2}{k-1} (2^k - 1) C_k C_{n-k} \tag{47}$$

We note, that the number thus derived not only singles out the position of the ground state with the highest population, but also counts two topologies as distinct, where the minor states interchange their position in the graph. This view is appropriate in situations, where not only the ground state, but also all excited states can be labeled, e.g. by their chemical shift, as derived from off-resonance experiments (CEST, off-resonance $R_{1\rho}$). In contrast, it has been argued that only the ground state is specified ('labeled') in the graph, which shifts the problem from testing more topologies to the problem of attributing the correct chemical shifts to the excited states [11].

The resultant numbers of different exchange topologies for n states (n vertices or nodes in graph theory) are given in table S1. For four states, all 38 possible labeled, connected graphs corresponding to unique topologies of 4-state exchange systems are given in Figure S10

# states	1	2	3	4	5	6
# topologies (fully labeled)	1	1	4	38	728	26704
# topologies (ground state labeled)	1	1	3	11	58	393

Table S1: Number of exchange topologies for n states, given by the number of connected, labeled graphs according to equation (47), and for topologies, in which only the ground state is labeled [11].

2 Supplementary Figures

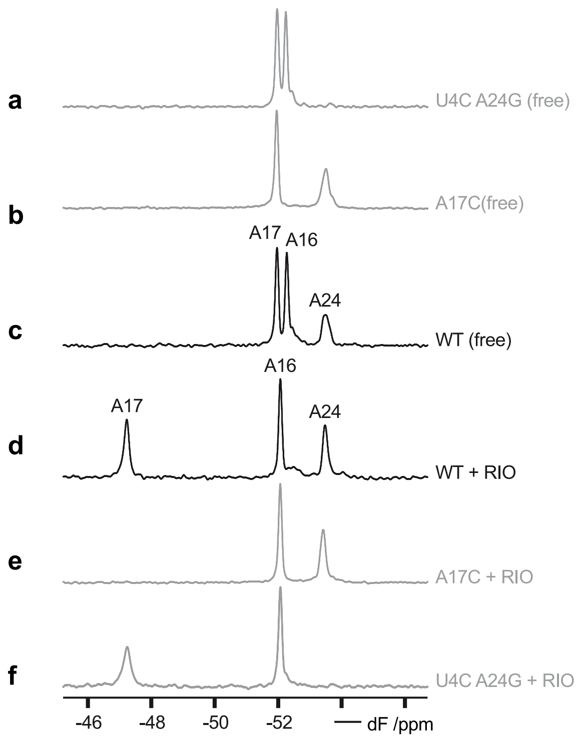

Figure S1: Assignment of ^{19}F resonances in the neomycin-sensing riboswitch. ^{19}F spectra of (a) the U4C A24G double mutant in the free riboswitch, (b) the A17C mutant in the free riboswitch, (c) the wild type sequence free riboswitch, (d) the wild type riboswitch in the presence of RIO, (e) the A17C mutant riboswitch in the presence of RIO and (f) the U4C A24G double mutant in the presence of RIO. Note that the A16 resonance is shifted in the A17C mutant. The upfield shoulder of the A16 resonance in the free riboswitch arises from a slow exchange process of A24. In addition, the resonance of A24 is broadened in the unbound riboswitch due to dynamics in the helix, which were not included in our analysis.

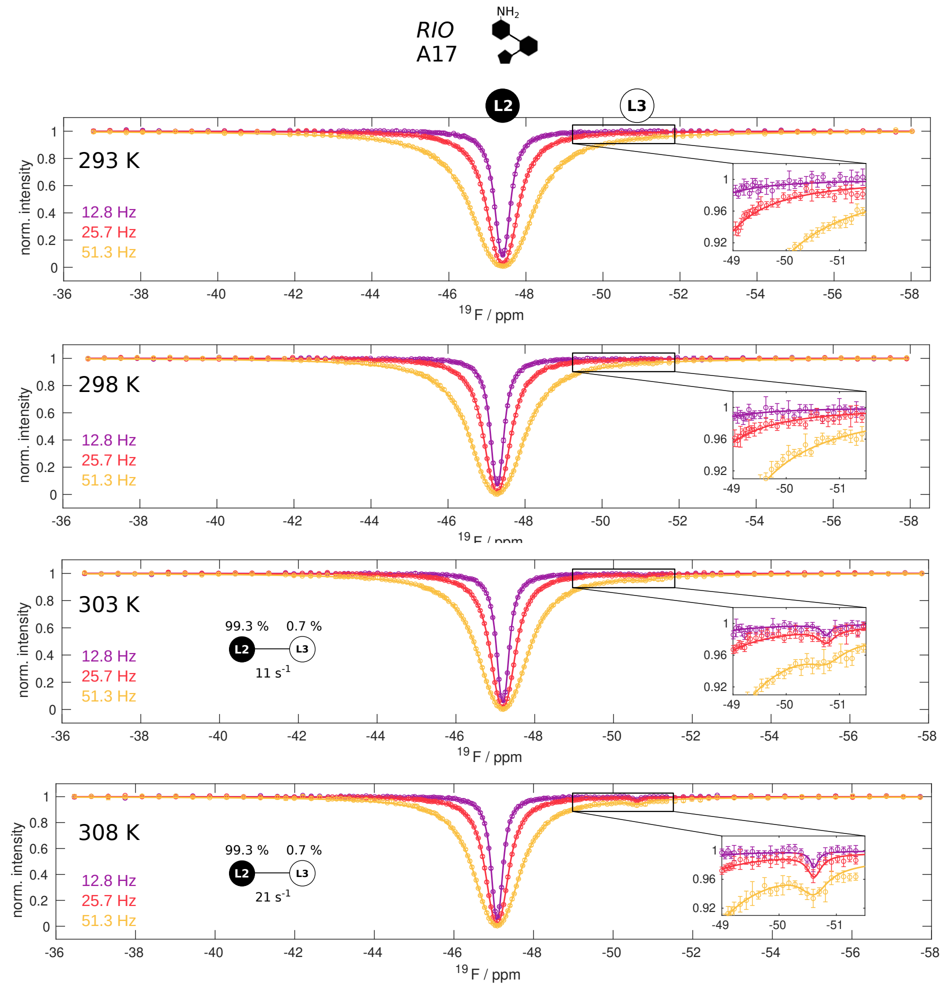

Figure S2: CEST data for the RIO-bound riboswitch at different temperatures. CEST profiles at 293 K, 298 K, 303 K and 308 K from A17 at B_1 fields of 12.8 Hz, 25.7 Hz and 51.3 Hz with data (circles with errorbars) and fits that use a model in the absence of exchange (293 K, 298 K) and a 2-state exchange model (303 K, 308 K) (solid line). The resonance positions of L2 and L3 are indicated above the CEST spectra. The insets show a zoom of the CEST profiles in the region of the L3. Values and uncertainties of the exchange processes are summarized in table S3.

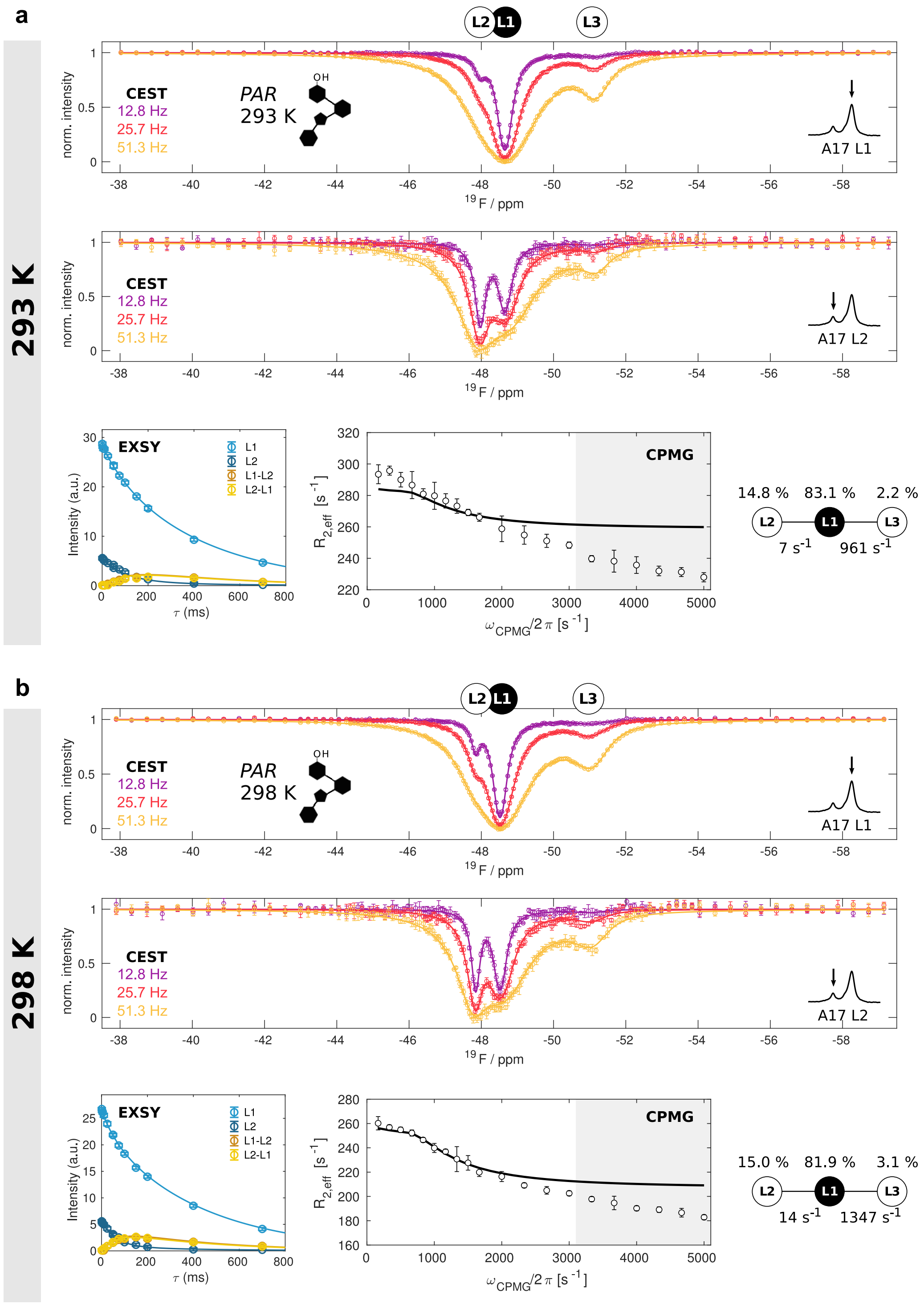

Figure S3: CEST, EXSY-exchange and CPMG data for the PAR-bound riboswitch.

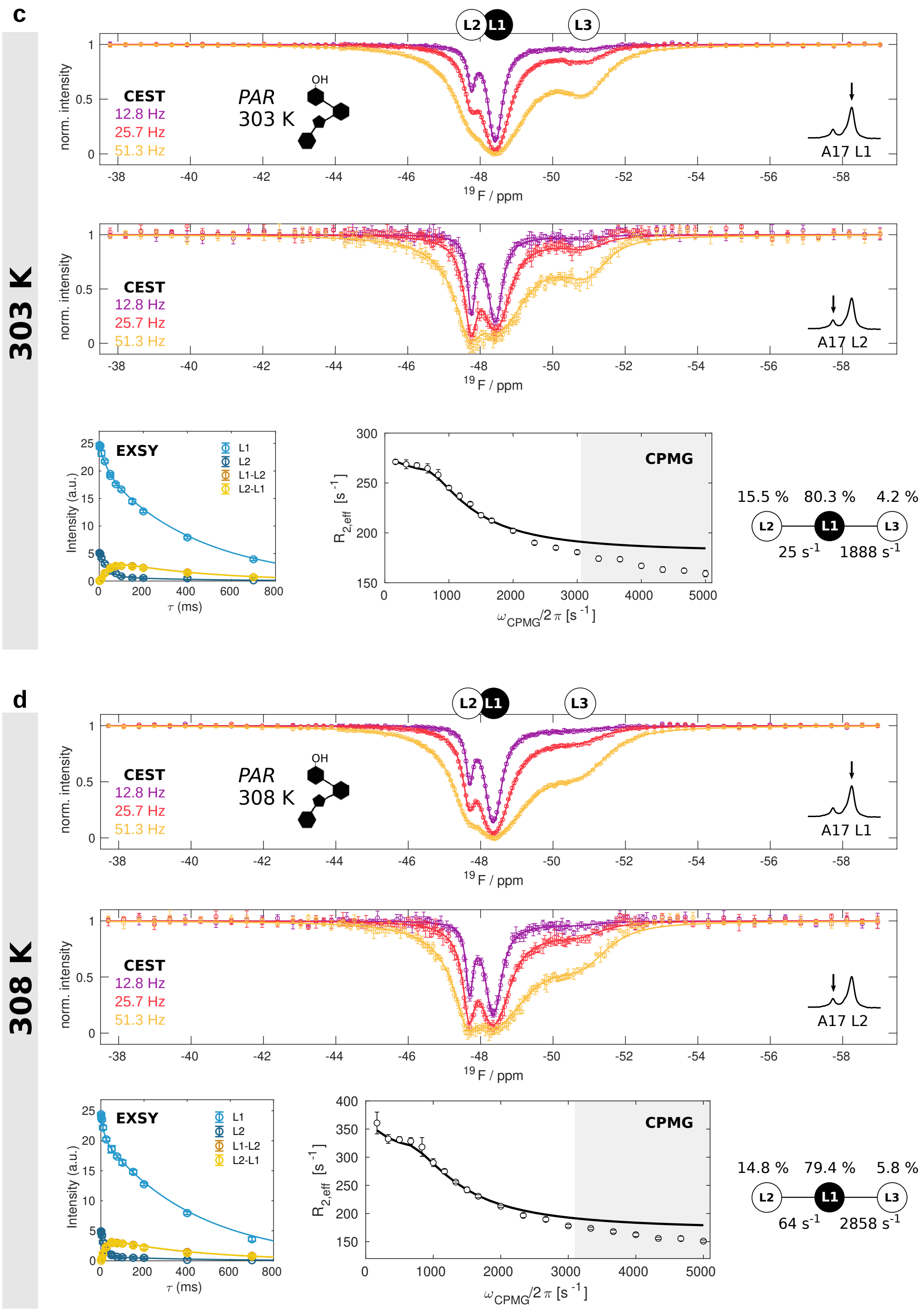

Figure S3: CEST, EXSY-exchange and CPMG data for the PAR-bound riboswitch (cont.). CEST profiles, EXSY-exchange data and CPMG curve of the A17F2 resonance at (a) 293 K, (b) 298 K, (c) 303 K and (d) 308 K, together with the parameters obtained by a least-square fitting routine. Values and uncertainties of the exchange processes are summarized in table S6.

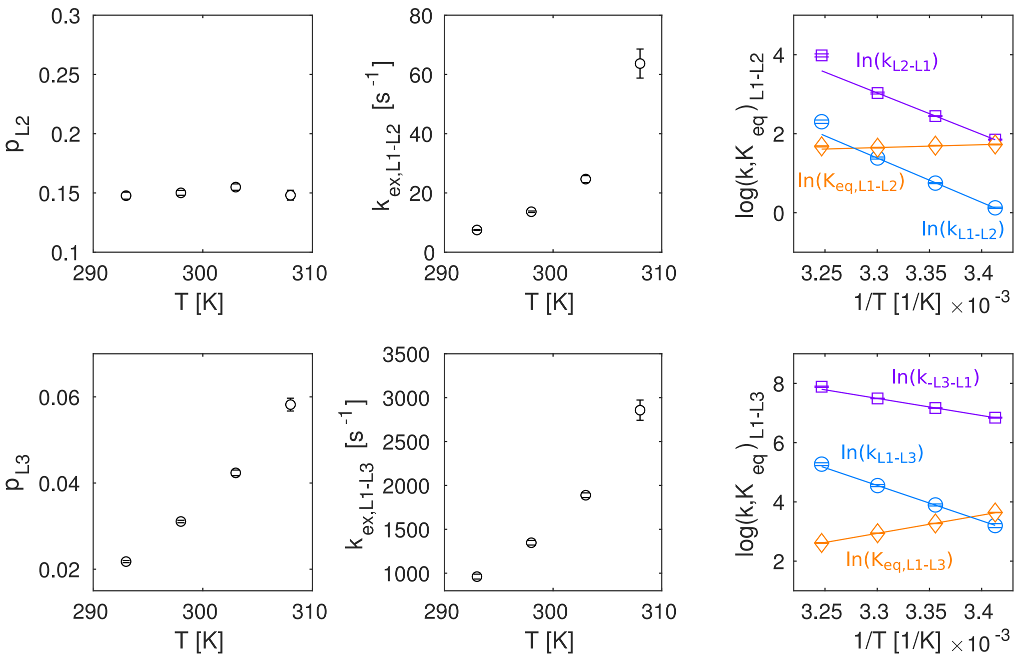

Figure S4: Temperature dependence of exchange processes in the PAR-bound riboswitch. Populations (p_{L2}, p_{L3}, left) and exchange rates ($k_{ex,L1-L2}$, $k_{ex,L1-L3}$, middle) as a function of temperature T. Right: Logarithmic rate constants k_{L1-L2}, k_{L1-L3}, k_{L2-L1} and k_{L3-L1} as well as equilibrium constants $K_{eq,L1-L2}$ and $K_{eq,L1-L3}$ as a function of inverse temperature T^{-1}. Values and uncertainties of the exchange processes are summarized in table S6, the extracted thermodynamic properties are summarized in table S9.

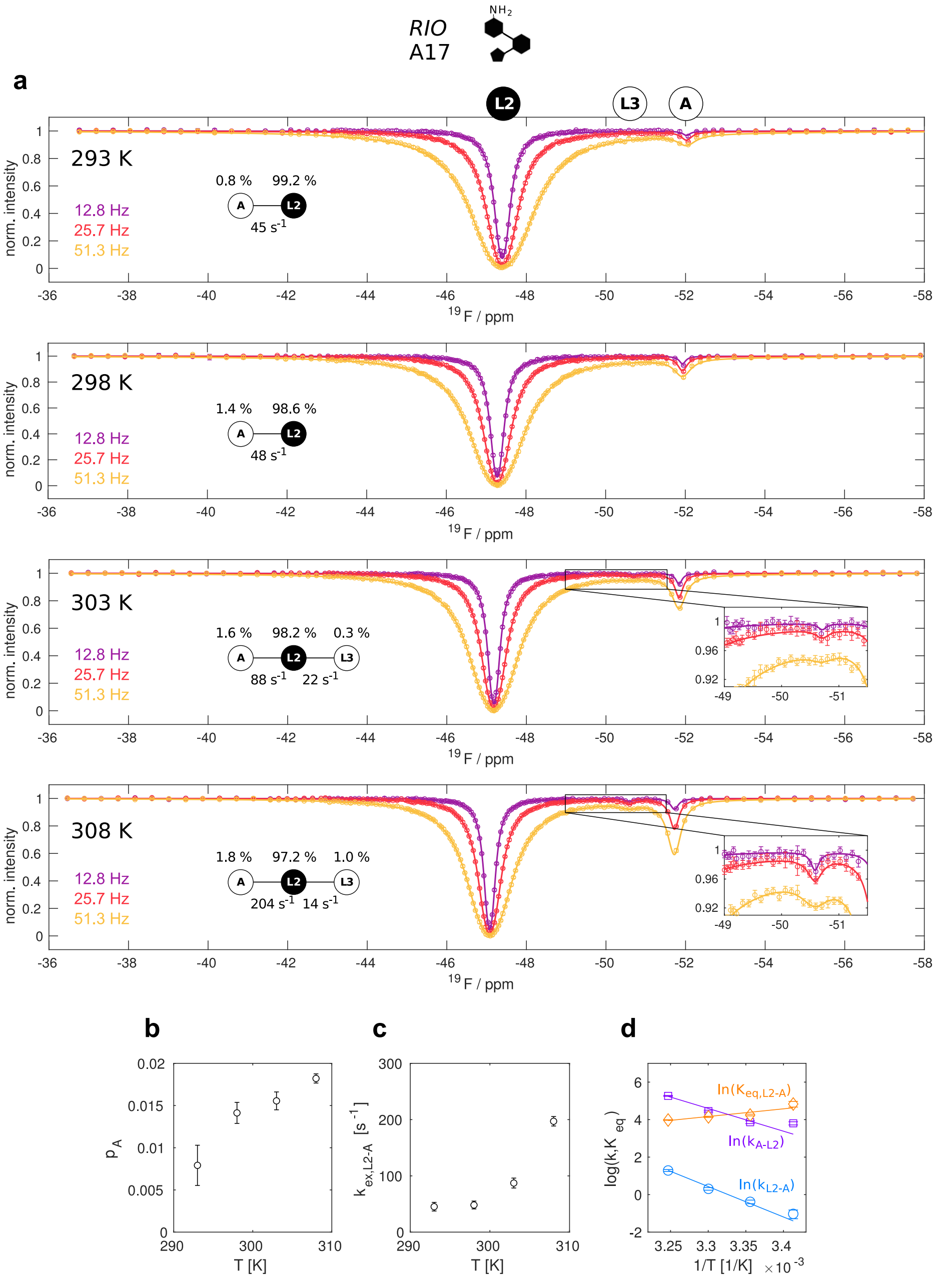

Figure S5: A three-state exchange in the unsaturated RIO-bound riboswitch. (a) CEST profiles at 293-308 K from A17 2F at B1 fields of 12.8 Hz, 25.7 Hz and 51.3 Hz with data (circles with errorbars) and the fit from the 2-state (293 K, 298 K) and 3-state (303 K, 308 K) exchange model (solid line). The resonance positions of L2, L3 and A are indicated above the CEST spectra. The insets show the CEST profiles in the region of the L3 state. (b) temperature dependent populations of the unbound riboswitch (p_A) (c) temperature dependent exchange rates between the L2 and A ($k_{ex,L2-A}$) (d) logarithmic plot of the rates k_{L2-A}, k_{A-L2} and $K_{eq,L2-A} = k_{L2-A}/k_{A-L2}$. Values and uncertainties of the exchange processes are summarized in table S4, the extracted thermodynamic properties are summarized in table S9.

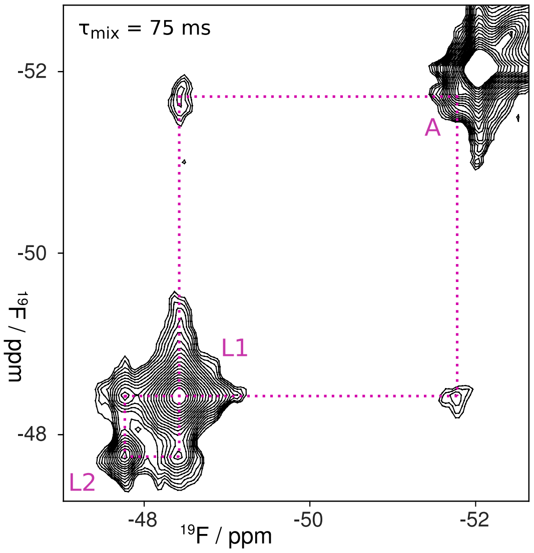

Figure S6: [^{19}F-^{19}F]-EXSY spectrum of the unsaturated PAR-bound riboswitch at 303 K with a mixing time of $\tau = 75$ ms. In addition to the L1-L2 cross peaks, weak L1-A cross peaks are visible. Note that the L1-A cross peaks were not used in the fitting of the data as the intensities are too low to be accurately quantified.

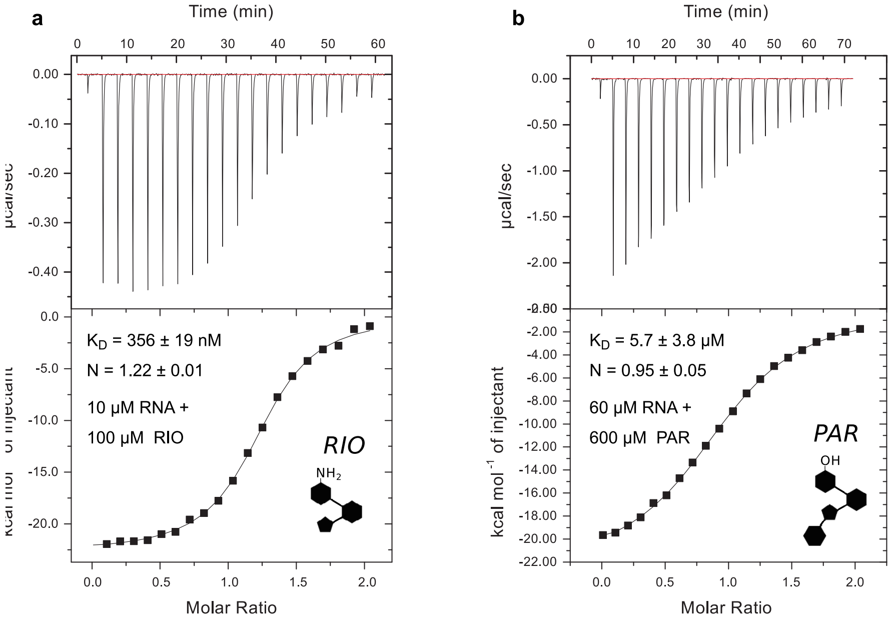

Figure S7: ITC titrations of the 2FA-labeled neomycin-binding riboswitch. ITC thermograms for ligand binding at 310 K show that the 2FA-labeled riboswitch retains a higher affinity towards RIO (a) compared to PAR (b)

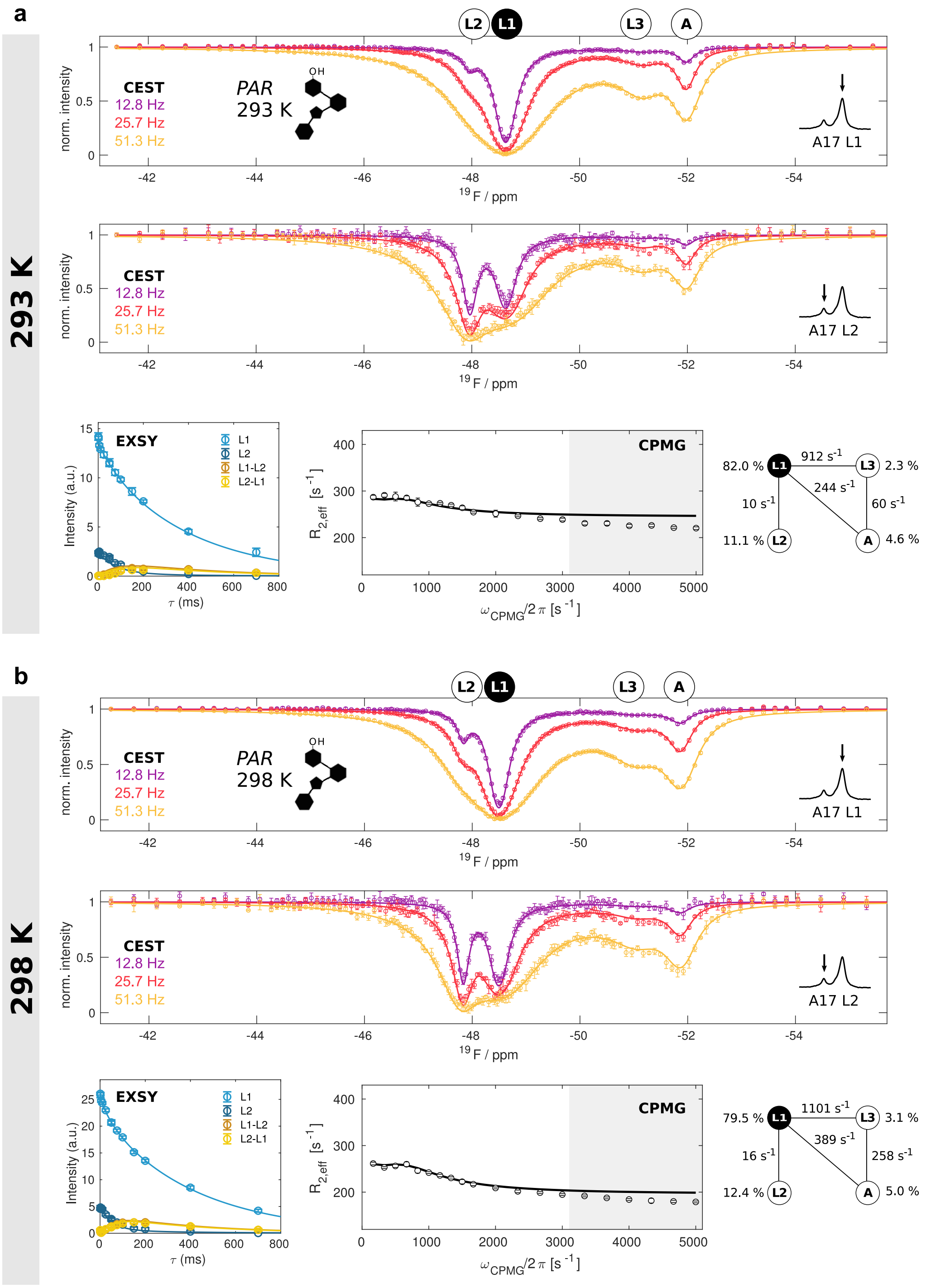

Figure S8: CEST, EXSY-exchange and CPMG data for the unsaturated PAR-bound riboswitch.

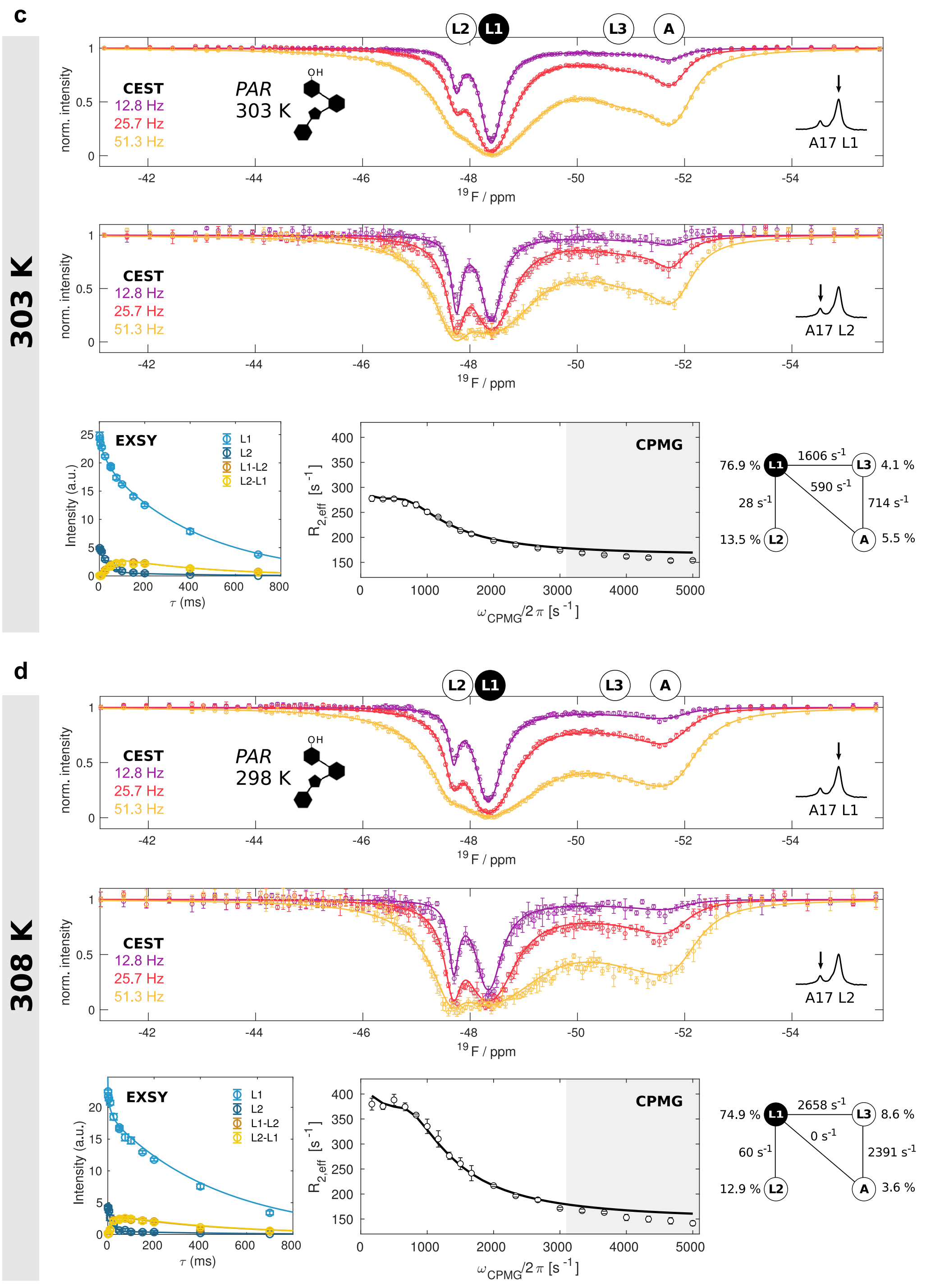

Figure S8: CEST, EXSY-exchange and CPMG data for the unsaturated PAR-bound riboswitch (cont.). CEST profiles, EXSY-exchange data and CPMG curve of the A17F2 resonance at (a) 293 K, (b) 298 K, (c) 303 K and (d) 308 K, together with the parameters obtained by a least-square fitting routine. Values and uncertainties of the exchange processes are summarized in table S7.

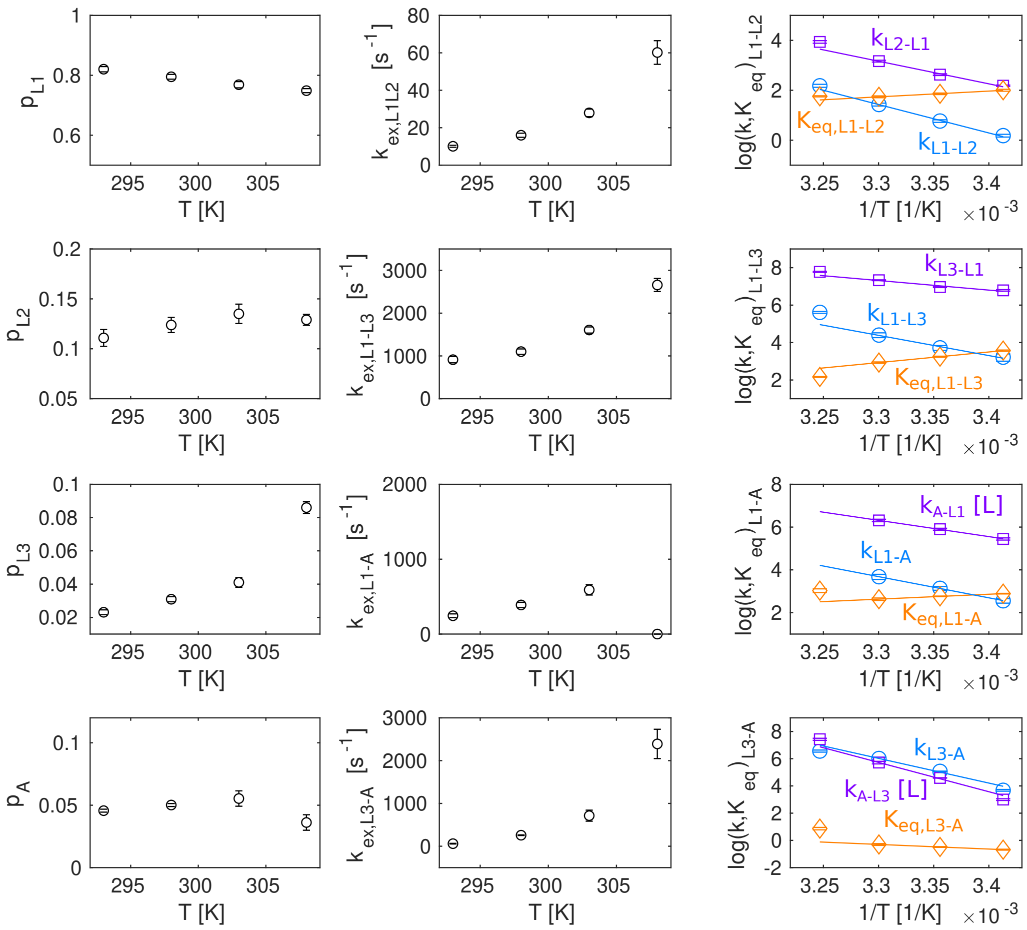

Figure S9: Temperature dependence of exchange processes in the unsaturated PAR-bound riboswitch. Populations (p_{L1}, p_{L2}, p_{L3}, p_{A}, left) and exchange rates ($k_{\text{ex},L1-L2}$, $k_{\text{ex},L1-L3}$, $k_{\text{ex},L1-A}$, $k_{\text{ex},L3-A}$, middle) as a function of temperature T. Right: Logarithmic rate constants k_{L1-L2}, k_{L1-L3}, k_{L1-A}, k_{L3-A}, k_{L2-L1}, k_{L3-L1}, k_{A-L1} and k_{A-L3} as well as equilibrium constants $K_{\text{eq},L1-L2}$, $K_{\text{eq},L1-L3}$, $K_{\text{eq},L1-A}$ and $K_{\text{eq},L3-A}$ as a function of inverse temperature T^{-1}. Values and uncertainties of the exchange processes are summarized in table S7, the extracted thermodynamic properties are summarized in table S9.

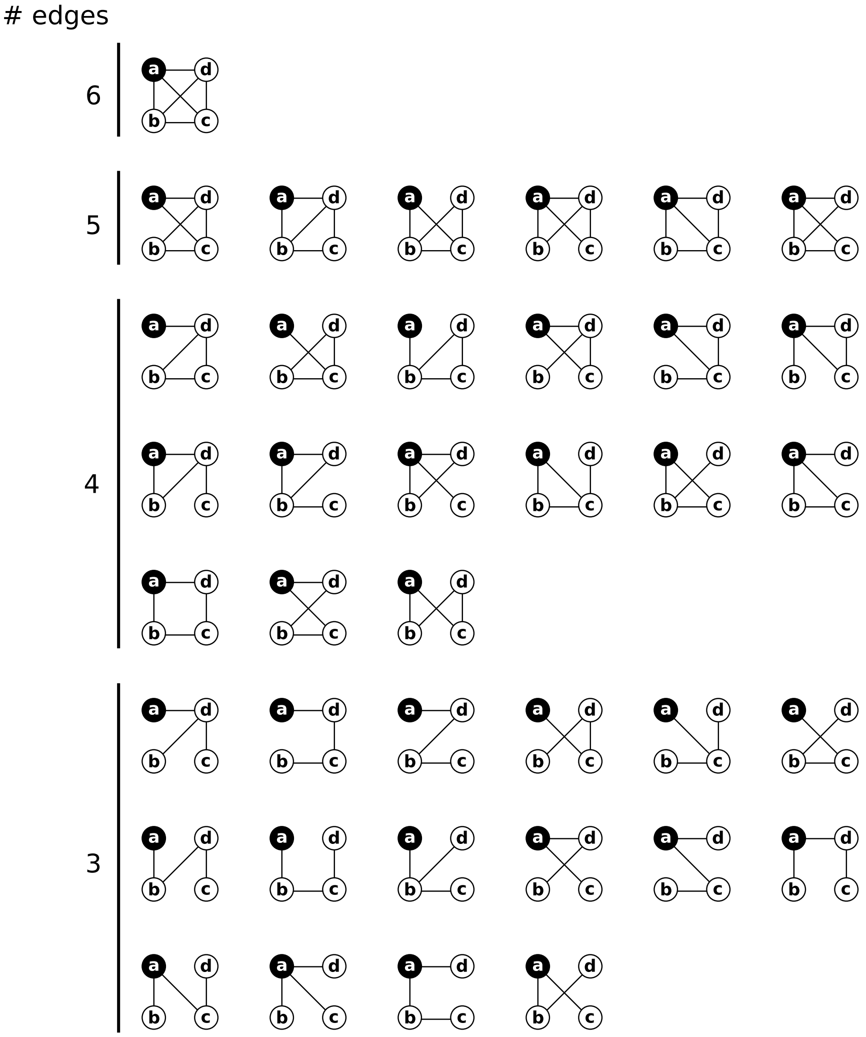

Figure S10: Connected, labeled graphs with four nodes and three to six edges, corresponding to the unique topologies of a 4-state exchange system with states $i = \{a, b, c, d\}$ distinguishable (labeled) by their chemical shift ω_i

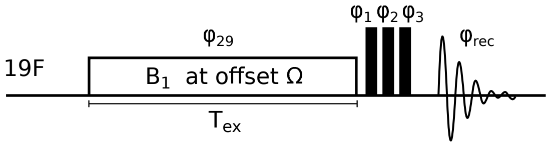

Figure S11: CEST experiment. Narrow rectangles indicate 90° pulses, which are applied along the x-axis unless indicated otherwise. The phase cycle is $\phi_1 = x$, $\phi_2 = [-x, x]$, $\phi_3 = [x, x, -x, -x, y, y, -y, -y]$, $\phi_{29} = [x]$, $\phi_{\mathrm{rec}} = [x, -x, -x, x, y, -y, -y, y]$.

3 Supplementary Tables

T [K]	model	p_{L1}	p_{L2}	$k_{ex,L1-L2}$ [s^{-1}]	R_1 [Hz]	R_2 [Hz]	ω_{L1} [ppm]	ω_{L2} [ppm]	χ^2	χ^2_ν	AIC	BIC	N	K	ΔAIC	ΔBIC	w_{AIC}	w_{BIC}	figure
303	1 state	-	-	-	2.72	286.1	-47.9395	-47.292	1499.6	2.42	-5503.3	-5481.1	624	5	124.8	120.4	0.00	0.00	2
303	2 state	0.675 ± 0.039	0.325 ± 0.039	0.29 ± 0.03	2.65 ± 0.01	282.6 ± 1.0	-47.941 ± 0.001	-47.2918 ± 0.001	1361.1	2.20	-5628.1	-5601.5	624	6	0.0	0.0	1.00	1.00	2

Table S2: Exchange parameters for the NEO-bound riboswitch neglecting or including exchange between L1 and L2 (main text figure 2). χ^2 is the error-weighted sum of squared residuals (eq. (36)), χ^2_ν is the reduced χ^2 (eq. (37)), AIC is the Akaike Information Criterion (eq. (39)), BIC is the Bayesion Information Criterion (eq. (38)), N is the total number of data points, K is the number of parameters in a model, ΔAIC and ΔBIC are the difference in AIC (BIC) of the current model compared to the model with a minimum AIC (BIC), and w_{AIC} and w_{BIC} are the model weights according to eqs. (40) and (41).

T [K]	model	p_{L2}	p_{L3}	$k_{ex,L1-L2}$ [s^{-1}]	R_1 [Hz]	R_2 [Hz]	ω_{L2} [ppm]	ω_{L3} [ppm]	χ^2	χ^2_ν	figure
303	2 state	0.993 ± 0.013	0.007 ± 0.013	10.5 ± 15.2	2.43 ± 0.04	148.3 ± 1.4	-47.1922 ± 0.00	-50.7552 ± 0.044	217.02	0.470	2, S2
308	2 state	0.993 ± 0.006	0.007 ± 0.006	20.8 ± 16.6	2.70 ± 0.05	131.1 ± 1.5	-47.0833 ± 0.00	-50.6147 ± 0.021	348.72	0.755	S2

Table S3: Exchange parameters for the RIO-bound riboswitch at 303 K and 308 K (main text figure 2, figure S2). χ^2 is the error-weighted sum of squared residuals (eq. (36)), χ^2_ν is the reduced χ^2 (eq. (37)).

T [K]	model	p_{L2}	p_A	p_{L3}	$k_{ex,L2-A}$	$k_{ex,L2-L3}$	$k_{ex,L3-A}$	R_1 [Hz]	R_2 [Hz]	ω_{L2} [ppm]	ω_A [ppm]	ω_{L3} [ppm]	figure
293	2 state	0.992 ± 0.002	0.008 ± 0.002	-	45.2 ± 7.7	-	-	2.34 ± 0.04	225.8 ± 2.1	-47.395 ± 0.001	-52.023 ± 0.01	-	S5
298	2 state	0.986 ± 0.003	0.014 ± 0.003	-	48.2 ± 7.0	-	-	2.57 ± 0.05	182.4 ± 2.0	-47.276 ± 0.001	-51.931 ± 0.006	-	S5
303	3 state	0.982 ± 0.001	0.016 ± 0.001	0.003 ± 0.001	87.2 ± 8.2	12.8 ± 5.1	11.6 ± 3.2	2.44 ± 0.04	142.5 ± 1.4	-47.181 ± 0.001	-51.836 ± 0.004	-50.708 ± 0.058	-
303	3 state linear (L3-L2-A)	0.982 ± 0.001	0.016 ± 0.001	0.003 ± 0.001	88.1 ± 8.8	21.8 ± 5.7	-	2.44 ± 0.05	142.4 ± 1.6	-47.181 ± 0.001	-51.836 ± 0.005	-50.711 ± 0.063	S5
303	3 state linear (L2-A-L3)	0.982 ± 0.001	0.016 ± 0.001	0.003 ± 0.001	87.7 ± 8.6	-	25.1 ± 6.4	2.44 ± 0.05	142.4 ± 1.6	-47.181 ± 0.001	-51.836 ± 0.005	-50.705 ± 0.056	-
308	3 state	0.973 ± 0.002	0.018 ± 0.001	0.009 ± 0.002	197.1 ± 13.7	0.0 ± 0.0	24.8 ± 3.8	2.50 ± 0.05	120.4 ± 1.3	-47.077 ± 0.001	-51.724 ± 0.004	-50.575 ± 0.028	-
308	3 state linear (L3-L2-A)	0.972 ± 0.002	0.018 ± 0.001	0.010 ± 0.002	203.9 ± 13.9	14.3 ± 2.4	-	2.49 ± 0.04	120.1 ± 1.3	-47.077 ± 0.001	-51.722 ± 0.004	-50.584 ± 0.020	S5
308	3 state linear (L2-A-L3)	0.973 ± 0.002	0.018 ± 0.001	0.009 ± 0.002	197.1 ± 13.0	-	24.8 ± 4.5	2.50 ± 0.05	120.4 ± 1.4	-47.077 ± 0.001	-51.724 ± 0.004	-50.575 ± 0.020	-

Table S4: Exchange parameters for the unsaturated RIO-bound riboswitch between 293 K and 308 K (figure S5).

T [K]	model	χ^2	χ^2_ν	AIC	BIC	N	K	ΔAIC	ΔBIC	w_{AIC}	w_{BIC}
293	2 state	484.57	1.05	-4735.6	-4710.7	468	6				
298	2 state	403.54	0.87	-4854.8	-4829.9	468	6				
303	3 state	265.67	0.58	-5091.6	-5050.1	468	10	1.50	5.64	0.21	0.03
303	3 state linear (L3-L2-A)	265.99	0.58	-5093.1	-5055.8	468	9	0.00	0.00	0.45	0.56
303	3 state linear (L2-A-L3)	266.29	0.58	-5092.5	-5055.2	468	9	0.60	0.60	0.34	0.41
308	3 state	219.75	0.48	-5187.8	-5146.3	468	10	2.00	6.15	0.26	0.04
308	3 state linear (L3-L2-A)	220.56	0.48	-5182.2	-5144.9	468	9	7.51	7.51	0.02	0.02
308	3 state linear (L2-A-L3)	219.75	0.48	-5189.8	-5152.4	468	9	0.00	0.00	0.72	0.93

Table S5: Error analysis of exchange in the unsaturated RIO-bound riboswitch between 293 K and 308 K. χ^2 is the error-weighted sum of squared residuals (eq. (36)), χ^2_ν is the reduced χ^2 (eq. (37)), AIC is the Akaike Information Criterion (eq. (39)), BIC is the Bayesion Information Criterion (eq. (38)), N is the total number of data points, K is the number of parameters in a model, ΔAIC and ΔBIC are the difference in AIC (BIC) of the current model compared to the model with a minimum AIC (BIC), and w_{AIC} and w_{BIC} are the model weights according to eqs. (40) and (41).

T [K]	data used	model	p_{L1}	p_{L2}	p_{L3}	$k_{\mathrm{ex},L1-L2}$	$k_{\mathrm{ex},L1-L3}$	$k_{\mathrm{ex},L2-L3}$	R_1 [Hz]	R_2 [Hz]	ω_{L1} [ppm]	ω_{L2} [ppm]	ω_{L3} [ppm]	figure
293	CEST, EXSY, CPMG	3 state linear (L2-L1-L3)	0.831 ± 0.023	0.148 ± 0.003	0.022 ± 0.001	7.5 ± 0.2	961.1 ± 27.8	-	2.30 ± 0.02	258.8 ± 1.0	-48.651 ± 0.009	-47.976 ± 0.003	-51.206 ± 0.005	5, S3, S4
298	CEST, EXSY, CPMG	3 state linear (L2-L1-L3)	0.819 ± 0.002	0.150 ± 0.002	0.031 ± 0.001	13.7 ± 0.3	1347.0 ± 26.2	-	2.36 ± 0.02	207.0 ± 1.0	-48.513 ± 0.001	-47.846 ± 0.002	-51.106 ± 0.005	5, S3, S4
303	CEST, EXSY, CPMG	3 state linear (L2-L1-L3)	0.803 ± 0.003	0.155 ± 0.003	0.042 ± 0.001	24.7 ± 1.2	1888.4 ± 26.8	-	2.31 ± 0.04	180.3 ± 2.1	-48.403 ± 0.005	-47.760 ± 0.055	-51.018 ± 0.006	5, S3, S4
303	CEST, EXSY, CPMG (L1,L2), $R^{\mathrm{off}}_{1\rho}$, $R^{\mathrm{on}}_{1\rho}$	3 state	0.801 ± 0.001	0.156 ± 0.001	0.042 ± 0.001	23.1 ± 0.4	2001.0 ± 13.8	0.743 ± 0.089	2.30 ± 0.01	176.1 ± 0.4	-48.404 ± 0.001	-47.759 ± 0.001	-51.070 ± 0.004	-
303	CEST, EXSY, CPMG (L1,L2), $R^{\mathrm{off}}_{1\rho}$, $R^{\mathrm{on}}_{1\rho}$	3 state linear (L2-L1-L3)	0.801 ± 0.001	0.156 ± 0.001	0.042 ± 0.001	23.3 ± 0.4	2001.4 ± 13.1	-	2.30 ± 0.01	176.1 ± 0.4	-48.404 ± 0.001	-47.759 ± 0.001	-51.070 ± 0.004	3
308	CEST, EXSY, CPMG	3 state linear (L2-L1-L3)	0.794 ± 0.005	0.148 ± 0.004	0.058 ± 0.002	63.7 ± 4.9	2857.5 ± 115.4	-	2.29 ± 0.04	171.0 ± 3.8	-48.326 ± 0.017	-47.694 ± 0.069	-50.907 ± 0.010	5, S3, S4

Table S6: Exchange parameters for the PAR-bound riboswitch between 293 K and 308 K (main text figure 3, figure S3)

T [K]	data used	model	p_{L1}	p_{L2}	p_{L3}	p_A	$k_{ex,L1-L2}$	$k_{ex,L1-L3}$	$k_{ex,L2-L3}$	$k_{ex,L1-A}$	$k_{ex,L2-A}$	$k_{ex,L3-A}$	R_1 [Hz]	R_2 [Hz]	ω_{L1} [ppm]	ω_{L2} [ppm]	ω_{L3} [ppm]	ω_A [ppm]	figure
293	CEST, EXSY, CPMG	tadpole 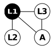	0.820 ± 0.008	0.111 ± 0.008	0.023 ± 0.001	0.046 ± 0.001	10.1 ± 0.5	912.4 ± 73.3	-	244.5 ± 24.7	-	59.7 ± 7.6	2.52 ± 0.06	245.0 ± 3.2	-48.626 ± 0.002	-47.966 ± 0.005	-51.197 ± 0.025	-51.981 ± 0.004	5, S8, S9
298	CEST, EXSY, CPMG	tadpole 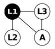	0.795 ± 0.008	0.124 ± 0.008	0.031 ± 0.002	0.050 ± 0.001	16.0 ± 1.1	1100.5 ± 62.8	-	388.7 ± 35.3	-	257.6 ± 7.2	2.45 ± 0.05	195.7 ± 2.5	-48.493 ± 0.002	-47.836 ± 0.003	-51.072 ± 0.024	-51.879 ± 0.005	5, S8, S9
303	CEST, EXSY, CPMG	tadpole 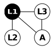	0.769 ± 0.010	0.135 ± 0.010	0.041 ± 0.003	0.055 ± 0.006	27.9 ± 2.0	1605.8 ± 71.5	-	590.3 ± 69.1	-	714.0 ± 128.9	2.36 ± 0.04	163.8 ± 2.7	-48.392 ± 0.002	-47.749 ± 0.004	-50.925 ± 0.044	-51.788 ± 0.016	5, S8, S9
303	CEST, EXSY, CPMG (L1,L2), $R_{1\rho}^{\mathrm{off}}$, $R_{1\rho}^{\mathrm{on}}$	complete 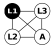	0.768 ± 0.007	0.136 ± 0.006	0.037 ± 0.002	0.059 ± 0.002	27.7 ± 1.3	1541.7 ± 42.8	0.0 ± 0.1	647.8 ± 30.0	0.0 ± 0.1	691.9 ± 75.9	2.36 ± 0.02	166.5 ± 1.0	-48.397 ± 0.001	-47.750 ± 0.003	-50.857 ± 0.032	-51.780 ± 0.008	-
303	CEST, EXSY, CPMG (L1,L2), $R_{1\rho}^{\mathrm{off}}$, $R_{1\rho}^{\mathrm{on}}$	diamond 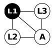	0.769 ± 0.008	0.136 ± 0.008	0.037 ± 0.002	0.059 ± 0.002	27.7 ± 1.7	1542.6 ± 50.8	-	648.3 ± 33.6	0.0 ± 0.1	690.9 ± 83.9	2.36 ± 0.02	166.5 ± 1.0	-48.396 ± 0.001	-47.750 ± 0.004	-50.858 ± 0.034	-51.780 ± 0.009	-
303	CEST, EXSY, CPMG (L1,L2), $R_{1\rho}^{\mathrm{off}}$, $R_{1\rho}^{\mathrm{on}}$	tadpole 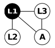	0.768 ± 0.006	0.136 ± 0.006	0.037 ± 0.002	0.059 ± 0.002	27.6 ± 1.7	1541.2 ± 45.5	-	647.9 ± 33.3	-	691.5 ± 76.3	2.36 ± 0.02	166.5 ± 0.9	-48.397 ± 0.001	-47.750 ± 0.003	-50.856 ± 0.031	-51.779 ± 0.009	4
303	CEST, EXSY, CPMG (L1,L2), $R_{1\rho}^{\mathrm{off}}$, $R_{1\rho}^{\mathrm{on}}$	tadpole 2 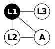	0.774 ± 0.005	0.133 ± 0.005	0.032 ± 0.001	0.062 ± 0.001	27.8 ± 1.4	1461.9 ± 45.8	-	796.8 ± 23.5	0.0 ± 0.1	-	2.32 ± 0.02	164.9 ± 0.9	-48.397 ± 0.001	-47.749 ± 0.003	-50.908 ± 0.023	-51.735 ± 0.005	-
303	CEST, EXSY, CPMG (L1,L2), $R_{1\rho}^{\mathrm{off}}$, $R_{1\rho}^{\mathrm{on}}$	starlike 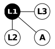	0.773 ± 0.002	0.133 ± 0.002	0.032 ± 0.001	0.062 ± 0.001	27.7 ± 0.6	1461.2 ± 19.0	-	793.9 ± 10.6	-	-	2.32 ± 0.01	165.0 ± 0.5	-48.397 ± 0.001	-47.749 ± 0.001	-50.909 ± 0.012	-51.735 ± 0.003	-
308	CEST, EXSY, CPMG	tadpole 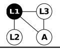	0.749 ± 0.007	0.129 ± 0.006	0.086 ± 0.04	0.036 ± 0.006	60.2 ± 6.3	2657.8 ± 152.2	-	0.0 ± 0.0	-	2391.1 ± 342.4	2.09 ± 0.05	148.3 ± 3.2	-48.312 ± 0.003	-47.695 ± 0.002	-51.074 ± 0.106	-51.891 ± 0.022	5, S8, S9

Table S7: Exchange parameters for the unsaturated PAR-bound riboswitch between 293 K and 308 K (main text figure 4, figure S8)

T [K]	data used	model		χ^2	χ^2_ν	AIC	BIC	N	K	ΔAIC	ΔBIC	w_{AIC}	w_{BIC}	figure
293	CEST, EXSY, CPMG	tadpole	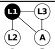	2046.5	2.30	768.3	840.4	906	15	-	-	-	-	5, S8, S9
298	CEST, EXSY, CPMG	tadpole	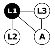	1994.9	2.24	745.1	817.3	906	15	-	-	-	-	5, S8, S9
303	CEST, EXSY, CPMG	tadpole	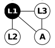	1615.5	1.81	554.0	626.1	906	15	-	-	-	-	5, S8, S9
303	CEST, EXSY, CPMG (L1,L2), $R_{1\rho}^{\text{off}}$, $R_{1\rho}^{\text{on}}$	complete	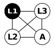	2792.8	1.87	961.4	1051.9	1513	17	3.99	14.64	0.09	0.00	-
303	CEST, EXSY, CPMG (L1,L2), $R_{1\rho}^{\text{off}}$, $R_{1\rho}^{\text{on}}$	diamond	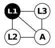	2792.9	1.87	959.4	1044.6	1513	16	2.03	7.36	0.24	0.02	-
303	CEST, EXSY, CPMG (L1,L2), $R_{1\rho}^{\text{off}}$, $R_{1\rho}^{\text{on}}$	tadpole	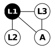	2792.8	1.86	957.4	1037.2	1513	15	0.00	0.00	0.67	0.97	4
303	CEST, EXSY, CPMG (L1,L2), $R_{1\rho}^{\text{off}}$, $R_{1\rho}^{\text{on}}$	tadpole 2	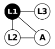	2924.0	1.95	1026.9	1106.7	1513	15	69.45	69.45	0.00	0.00	-
303	CEST, EXSY, CPMG (L1,L2), $R_{1\rho}^{\text{off}}$, $R_{1\rho}^{\text{on}}$	tadpole	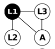	2923.7	1.95	1024.7	1099.2	1513	14	67.29	61.96	0.00	0.00	-
308	CEST, EXSY, CPMG	tadpole	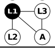	1552.6	1.74	518.0	590.2	906	15	-	-	-	-	5, S8, S9

Table S8: Error analysis of exchange in the unsaturated PAR-bound riboswitch between 293 K and 308 K. χ^2 is the error-weighted sum of squared residuals (eq. (36)), χ^2_ν is the reduced χ^2 (eq. (37)), AIC is the Akaike Information Criterion (eq. (39)), BIC is the Bayesion Information Criterion (eq. (38)), N is the total number of data points, K is the number of parameters in a model, ΔAIC and ΔBIC are the difference in AIC (BIC) of the current model compared to the model with a minimum AIC (BIC), and w_{AIC} and w_{BIC} are the model weights according to eqs. (40) and (41).

	exchange	ΔG (kcal mol^{-1})	ΔH (kcal mol^{-1})	$T\Delta S$ (kcal mol^{-1})	$\Delta G^{\ddagger}_{\rightarrow}$ (kcal mol^{-1})	$\Delta H^{\ddagger}_{\rightarrow}$ (kcal mol^{-1})	$T\Delta S^{\ddagger}_{\rightarrow}$ (kcal mol^{-1})	$\Delta G^{\ddagger}_{\leftarrow}$ (kcal mol^{-1})	$\Delta H^{\ddagger}_{\leftarrow}$ (kcal mol^{-1})	$T\Delta S^{\ddagger}_{\leftarrow}$ (kcal mol^{-1})	figure
unsaturated RIO-bound	L2-A	2.54 ± 0.05	8.53 ± 2.13	5.99 ± 2.08	9.53 ± 0.08	32.88 ± 3.22	23.35 ± 3.14	7.00 ± 0.06	24.36 ± 2.33	17.36 ± 2.27	S5
saturated PAR-bound	L1-L2	1.00 ± 0.01	1.47 ± 0.04	0.47 ± 0.03	17.05 ± 0.03	21.93 ± 0.64	4.87 ± 0.67	16.05 ± 0.03	20.46 ± 0.64	4.41 ± 0.67	5, S4
	L1-L3	1.78 ± 0.01	12.33 ± 0.12	10.55 ± 0.13	15.13 ± 0.01	23.30 ± 0.29	8.18 ± 0.28	13.34 ± 0.01	10.97 ± 0.25	-2.37 ± 0.25	5, S4
unsaturated PAR-bound	L1-L2	1.06 ± 0.05	4.64 ± 0.03	3.58 ± 0.02	17.04 ± 0.07	22.18 ± 0.33	5.15 ± 0.37	15.98 ± 0.05	17.54 ± 0.32	1.56 ± 0.37	5, S9
	L1-L3	1.878 ± 0.04	11.38 ± 0.26	9.60 ± 0.30	15.23 ± 0.05	21.04 ± 0.63	5.81 ± 0.61	13.45 ± 0.03	9.66 ± 0.65	-3.79 ± 0.63	5, S9
	L1-A	1.59 ± 0.07	4.69 ± 1.76	3.10 ± 1.83	15.66 ± 0.09	19.24 ± 1.75	3.57 ± 1.83	14.07 ± 0.07	14.55 ± 0.46	0.48 ± 0.52	5, S9
	L3-A	-0.18 ± 0.07	-6.78 ± 1.01	-6.60 ± 1.08	14.25 ± 0.08	35.30 ± 2.80	21.05 ± 2.88	14.43 ± 0.10	42.08 ± 2.90	27.65 ± 2.99	5, S9

Table S9: Summary of thermodynamic parameters for the three-site and four-site exchange in the unsaturated RIO-bound riboswitch and the saturated and unsaturated PAR-bound riboswitch at 303 K

4 Supplementary References

[1] J. SantaLucia, L. X. Shen, Z. Cai, H. Lewis, and I. Tinoco, "Synthesis and NMR of RNA with Selective isotopic enrichment in the bases," *Nucleic Acids Research*, vol. 23, no. 23, pp. 4913–4921, 1995.

[2] F. Delaglio, S. Grzesiek, G. Vuister, G. Zhu, J. Pfeifer, and A. Bax, "NMRPipe: A multidimensional spectral processing system based on UNIX pipes," *Journal of Biomolecular NMR*, vol. 6, Nov. 1995.

[3] M. Guenneugues, P. Berthault, and H. Desvaux, "A Method for DeterminingB1Field Inhomogeneity. Are the Biases Assumed in Heteronuclear Relaxation Experiments Usually Underestimated?," *Journal of Magnetic Resonance*, vol. 136, pp. 118–126, Jan. 1999.

[4] J. H. Overbeck, W. Kremer, and R. Sprangers, "A suite of 19F based relaxation dispersion experiments to assess biomolecular motions," *Journal of Biomolecular NMR*, vol. 74, pp. 753–766, Dec. 2020.

[5] P. Vallurupalli, G. Bouvignies, and L. E. Kay, "Studying "Invisible" Excited Protein States in Slow Exchange with a Major State Conformation," *Journal of the American Chemical Society*, vol. 134, pp. 8148–8161, May 2012.

[6] O. Trott and A. G. Palmer, "R 1ρ relaxation outside of the fast-exchange limit," *Journal of Magnetic Resonance*, vol. 154, no. 1, pp. 157–160, 2002.

[7] M. Rance and A. G. Palmer, "Compact expressions for R1 relaxation for N-site chemical exchange using Schur decomposition," *Journal of Magnetic Resonance*, vol. 313, p. 106705, Apr. 2020.

[8] D. M. Korzhnev, V. Y. Orekhov, and L. E. Kay, "Off-resonance R1ρ NMR studies of exchange dynamics in proteins with low spin-lock fields: An application to a fyn SH3 domain," *Journal of the American Chemical Society*, vol. 127, no. 2, pp. 713–721, 2005.

[9] E.-J. Wagenmakers and S. Farrell, "AIC model selection using Akaike weights," *Psychonomic Bulletin & Review*, vol. 11, pp. 192–196, Feb. 2004.

[10] A. Rangadurai, E. S. Szymaski, I. J. Kimsey, H. Shi, and H. M. Al-Hashimi, "Characterizing micro-to-millisecond chemical exchange in nucleic acids using off-resonance R1ρ relaxation dispersion," *Progress in Nuclear Magnetic Resonance Spectroscopy*, vol. 112-113, pp. 55–102, June 2019.

[11] F.-A. Chao, Y. Zhang, and R. A. Byrd, "Theoretical classification of exchange geometries from the perspective of NMR relaxation dispersion," *Journal of Magnetic Resonance*, vol. 328, p. 107003, July 2021.

[12] A. Nijenhuis and H. S. Wilf, "The enumeration of connected graphs and linked diagrams," *Journal of Combinatorial Theory, Series A*, vol. 27, pp. 356–359, Nov. 1979.
